# Osteoclast-derived exosomal miR-214-3p inhibits osteoblastic bone formation

**DOI:** 10.1038/ncomms10872

**Published:** 2016-03-07

**Authors:** Defang Li, Jin Liu, Baosheng Guo, Chao Liang, Lei Dang, Cheng Lu, Xiaojuan He, Hilda Yeuk-Siu Cheung, Liang Xu, Changwei Lu, Bing He, Biao Liu, Atik Badshah Shaikh, Fangfei Li, Luyao Wang, Zhijun Yang, Doris Wai-Ting Au, Songlin Peng, Zongkang Zhang, Bao-Ting Zhang, Xiaohua Pan, Airong Qian, Peng Shang, Lianbo Xiao, Baohong Jiang, Chris Kong-Chu Wong, Jiake Xu, Zhaoxiang Bian, Zicai Liang, De-an Guo, Hailong Zhu, Weihong Tan, Aiping Lu, Ge Zhang

**Affiliations:** 1 Institute for Advancing Translational Medicine in Bone and Joint Diseases, School of Chinese Medicine, Hong Kong Baptist University, Hong Kong SAR 999077, China; 2 Institute of Integrated Bioinfomedicine and Translational Science, School of Chinese Medicine, Hong Kong Baptist University, Hong Kong SAR 999077, China; 3 Shenzhen Lab of Combinatorial Compounds and Targeted Drug Delivery, HKBU Institute of Research and Continuing Education, Shenzhen 518057, China; 4 Research Group of Bone and Joint Diseases, HKBU Institute of Science and Technology, Haimen 226100, China; 5 Academician Chen Xinzi Workroom for Advancing Translational Medicine in Bone and Joint Diseases, Kunshan RNAi Institute, Kunshan Industrial Technology Research Institute, Kunshan, Jiangsu 215300, China; 6 Shum Yiu Foon Shum Bik Chuen Memorial Centre for Cancer and Inflammation Research, Hong Kong Baptist University, Hong Kong SAR 999077, China; 7 Hong Kong Baptist University Branch of State Key Laboratory of Chemo/Biosensing and Chemometrics of Hunan University, Hong Kong 999077, China; 8 Hong Kong Baptist University–Northwestern Polytechnical University Joint Research Centre for Translational Medicine on Musculoskeletal Health in Space, Shenzhen 518057, China; 9 Institute of Basic Research in Clinical Medicine, China Academy of Chinese Medical Sciences, Beijing 100700, China; 10 Department of Biology and Chemistry, City University of Hong Kong, Hong Kong SAR 999077, China; 11 Department of Spine Surgery, Shenzhen People's Hospital, Ji Nan University Second College of Medicine, Shenzhen 518020, China; 12 School of Chinese Medicine, Faculty of Medicine, Chinese University of Hong Kong, Hong Kong SAR 999077, China; 13 Department of Orthopaedics and Traumatology, Bao'an Hospital Affiliated to Southern Medical University and Shenzhen 8th People Hospital, Shenzhen 518100, China; 14 Key Laboratory for Space Bioscience and Biotechnology, Institute of Special Environmental Biophysics, School of Life Science, Northwestern Polytechnical University, Xi'an 710072, China; 15 Institute of Arthritis Research, Shanghai Academy of Chinese Medical Sciences, Shanghai 200052, China; 16 Shanghai Institute of Materia Medica, Chinese Academy of Sciences, Shanghai 201203, China; 17 Department of Biology, Hong Kong Baptist University, Hong Kong SAR 999077, China; 18 Molecular Laboratory, School of Pathology and Laboratory Medicine, University of Western Australia, Nedlands, Western Australia 6907, Australia

## Abstract

Emerging evidence indicates that osteoclasts direct osteoblastic bone formation. MicroRNAs (miRNAs) have a crucial role in regulating osteoclast and osteoblast function. However, whether miRNAs mediate osteoclast-directed osteoblastic bone formation is mostly unknown. Here, we show that increased osteoclastic miR-214-3p associates with both elevated serum exosomal miR-214-3p and reduced bone formation in elderly women with fractures and in ovariectomized (OVX) mice. Osteoclast-specific *miR-214-3p* knock-in mice have elevated serum exosomal miR-214-3p and reduced bone formation that is rescued by osteoclast-targeted antagomir-214-3p treatment. We further demonstrate that osteoclast-derived exosomal miR-214-3p is transferred to osteoblasts to inhibit osteoblast activity *in vitro* and reduce bone formation *in vivo*. Moreover, osteoclast-targeted miR-214-3p inhibition promotes bone formation in ageing OVX mice. Collectively, our results suggest that osteoclast-derived exosomal miR-214-3p transfers to osteoblasts to inhibit bone formation. Inhibition of miR-214-3p in osteoclasts may be a strategy for treating skeletal disorders involving a reduction in bone formation.

## Abstract (web summary)

In previous studies the authors discovered that miR-214 inhibits osteoblastic bone formation. Here they extend on these findings, using ovariectomized mice and samples from patients with bone fractures, to show that miR-214 is a mediator of osteoclast-osteoblast crosstalk.

## Graphical abstract

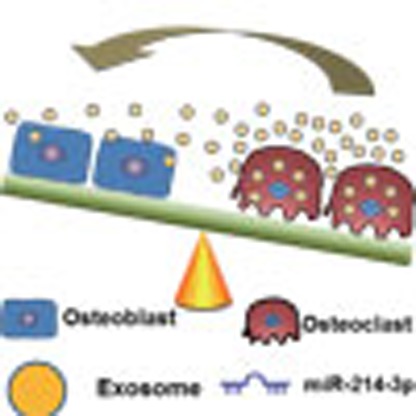

Bone is a dynamic tissue that undergoes life-long remodelling regulated by the tight coupling of bone resorption and bone formation^1^. The disruption of this equilibrium, that is, excessive bone resorption and/or reduced bone formation, will cause osteopenia and ultimately lead to osteoporosis and fracture especially in elderly individuals^2^,^3^. Bone remodelling is coordinately regulated by the bone-resorbing osteoclasts and bone-forming osteoblasts, highlighting the cross-talk between the two types of cells^3^,^4^. Aside from the well-documented regulatory mechanism of osteoblast-directed osteoclastic bone resorption, accumulating evidence indicates that osteoclasts in turn regulate osteoblastic bone formation either by direct cell–cell contact^5^,^6^ or indirectly via cytokines^7^. However, it is unclear whether there are other efficient ‘paracrine' approaches for the osteoclast-to-osteoblast communication.

MicroRNAs (miRNAs) are ∼22-nucleotide (nt) noncoding RNAs involved in the regulation of gene expression to coordinate a broad spectrum of biological processes^8^,^9^,^10^,^11^,^12^. A series of miRNAs has been characterized to regulate osteogenic activity and osteoblastic bone formation, and the dysregulation of these miRNAs has been linked with the skeletal disorders involving a reduction in bone formation^13^,^14^. Moreover, recent studies have demonstrated that miRNAs are presented in body fluid, for example, serum^14^,^15^,^16^, and they are transported in extracellular vesicles, for example, exosomes^17^,^18^,^19^,^20^, unveiling their novel function as extracellular signals between cells and their extracellular matrix. However, there is a lack of functional miRNAs identified as intercellular signals between osteoclasts and osteoblasts.

MiR-214-3p, a vertebrate-specific member of miRNA precursors, is reported to be involved in the regulation of hepatic gluconeogenesis^21^. MiR-214-3p also has a crucial role in skeletal disorders. MiR-214-3p has been shown to suppress osteogenic differentiation of C2C12 myoblast cells by targeting Osterix^22^, an osteoblast-specific transcription factor. Our previous study also identified that miR-214-3p could target ATF4, an important osteogenic transcriptional factor, to suppress bone formation^23^. Furthermore, miR-214-3p promotes osteoclastogenesis through PI3K/Akt pathway via targeting phosphatase and tensin homologue (PTEN)^24^.

Here, we examine the expression of bone metabolism-related miRNAs in bone specimens and serum exosomes from elderly bone-fracture women as well as ageing ovariectomized (OVX) mice and show that increased osteoclastic miR-214-3p level associates with both elevated serum exosomal miR-214-3p level and reduced bone formation. We present *in vitro* and *in vivo* evidence to demonstrate that osteoclast-derived exosomal miR-214-3p could transfer to osteoblasts to inhibit osteoblastic bone formation. Moreover, we show that osteoclast-targeted antagomir-214-3p treatment could promote bone formation in ageing OVX mice.

## Results

### High miR-214-3p in osteoclasts associates with reduced bone formation

**Figure 1:**
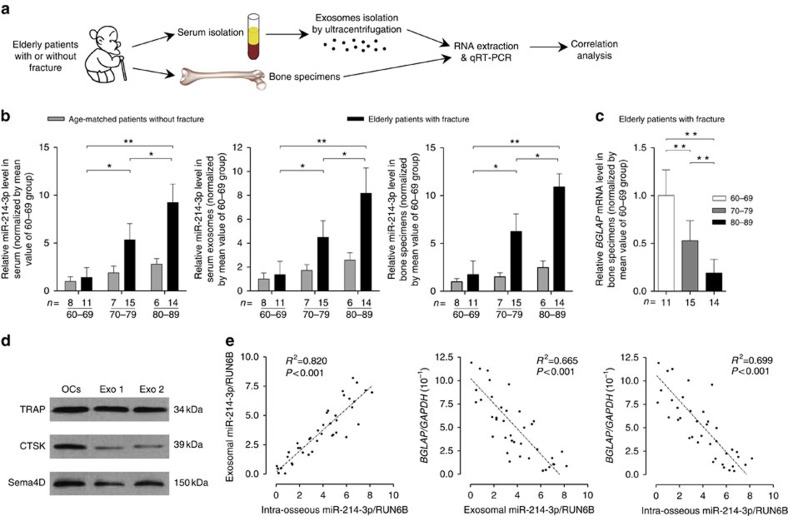
Increased intraosseous miR-214-3p associates with elevated serum exosomal miR-214-3p and reduced bone formation in elderly patients. (**a**) A schematic diagram illustrating the experimental design. Serum and bone samples were collected from elderly woman patients with or without fractures, and divided into 60–69, 70–79 and 80–89 groups, respectively, according to age. (**b**) Real-time PCR analysis of the age-related changes in miR-214-3p levels in whole serum (left), serum exosomes (middle) and bone specimens (right) from elderly patients with or without fractures, respectively. The relative miR-214-3p level in each group was normalized to the mean value of the 60–69 group. Human RUN6B was used as the internal control. All data are the mean±s.d. *P<0.05, **P<0.01. Two-way analysis of variance (ANOVA) with a *Turkey's* multiple comparisons test was performed. Both the time effect (age), the group effect (with or without fractures) and the time-by-group interaction effect were all statistically significant for all the examined parameters. (**c**) Real-time PCR analysis showing the age-related changes in *BGLAP* mRNA levels in bone specimens from elderly patients with fractures, respectively. The relative *BGLAP* mRNA level in each group was normalized to the mean value of the 60–69 group. Human *GAPDH* mRNA was used as the internal control. Data are the mean±s.d. **P<0.01. One-way ANOVA with a *post-hoc* test was performed. (**d**) Western blot analysis of the osteoclast marker proteins (CTSK, TRAcP5 and Sema4D) in the lysates of serum exosomes isolated from elderly patients with or without fractures. OCs, the lysates of osteoclasts differentiated from human peripheral blood mononuclear cells. Exo 1, the lysates of serum exosomes from elderly patients without fractures. Exo 2, the lysates of serum exosomes from elderly patients with fracture. (**e**) Correlation analysis between exosomal and intra-osseous miR-214-3p levels (left), between exosomal miR-214-3p level and intra-osseous *BGLAP* mRNA level (middle) and between intra-osseous miR-214-3p level and intra-osseous *BGLAP* mRNA level (right), respectively, in elderly patients with fractures. The *n* value for each group is indicated at the bottom of each histogram.

To search for candidate miRNAs potentially participating in mediating osteoclast-to-osteoblast communication, we selected 12 miRNAs that was previously reported to be involved in the regulation of bone metabolism^23^,^25^,^26^,^27^ (Supplementary Table 1). Thereafter, we examined the expression of those miRNAs in whole serum, serum exosomes and bone specimens collected from 40 elderly female patients (age range from 61 to 89 years) with low-energy fractures (Fig. 1a and Supplementary Tables 2 and 3) by real-time PCR. The serum exosomes were isolated from whole serum by ultracentrifugation and documented by the previously reported characteristics of exosomes^28^,^29^, including the diameter (a median size of 95 nm) and exosomal markers CD9, CD63 and FLOT1 (the cytoplasmic protein calnexin set as a negative control; Supplementary Fig. 1a,b). The real-time PCR analysis showed that only the level of miR-214-3p but not the other examined miRNAs increased with age in whole serum, serum exosomes and bone specimens, respectively (Fig. 1b and Supplementary Fig. 1c). Consistently, we also found an age-related increase in miR-214-3p level in whole serum, serum exosomes and bone tissues in the age-matched controls without fractures, respectively (Fig. 1b and Supplementary Tables 4 and 5). Interestingly, the age-related increase in miR-214-3p level was accelerated in those specimens from the above elderly patients with low-energy fractures (Fig. 1b). In consistent with a recent study showing that 3′ end-uridylated miRNA isoforms appear overrepresented in exosomes^30^, we also detected 3′ end-uridylated miR-214-3p in the above serum exosomes, suggesting that miR-214-3p was enriched in serum exosomes (Supplementary Fig. 1d). Moreover, the osteoclastic marker proteins (CTSK, TRAcP5 and Sema4D) were also detectable in serum exosomes by western blot analysis (Fig. 1c and Supplementary Fig. 17). On the other hand, we found that the mRNA level of bone formation marker gene *BGLAP* (osteocalcin) decreased with age in bone specimens from the above elderly patients with fractures (Fig. 1d). Interestingly, the miR-214-3p level was almost equal in whole serum and serum exosomes (Supplementary Fig. 2a,b). Furthermore, the serum exosomal miR-214-3p level was positively correlated with the intra-osseous miR-214-3p level (Fig. 1e), whereas we found a negative correlation between serum exosomal miR-214-3p level and intra-osseous *BGLAP* mRNA level and between intra-osseous miR-214-3p level and intra-osseous *BGLAP* mRNA level, respectively (Fig. 1e).

**Figure 2:**
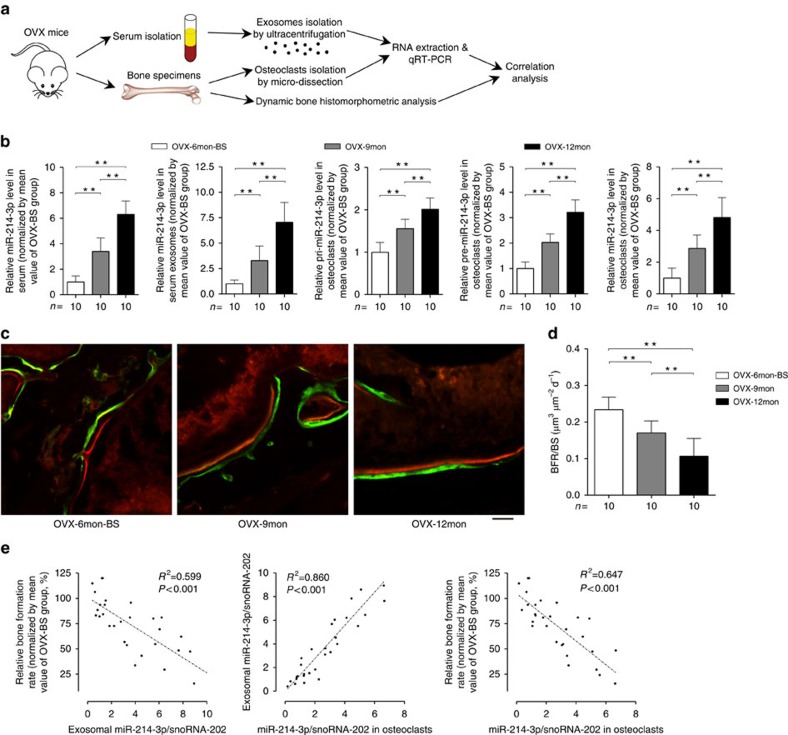
Increased intra-osteoclast miR-214-3p associates with elevated serum exosomal miR-214-3p and reduced bone formation in OVX mice. (**a**) A schematic diagram illustrating the experimental design. Serum and bone samples were collected from female C57BL/6 mice that were ovariectomized at 6-month-old and killed at 6 months (as baseline, OVX-6mon-BS), 9 months (OVX-9mon) and 12 months (OVX-12mon) after ovariectomy, respectively. (**b**) Real-time PCR analysis of the age-related changes in the levels of miR-214-3p in whole serum (left) and serum exosomes (middle left), and the levels of pri-miR-214-3p (middle), pre-miR-214-3p (middle right) and mature miR-214-3p in osteoclasts (right) in OVX mice in three age-subgroups, respectively. The relative miR-214-3p level in each group was normalized to the mean value of the OVX-BS group. Mouse snoRNA-202 was used as the internal control. (**c**) Representative images of new bone formation at distal femur metaphysis assessed by double labelling with calcein green and xylenol orange in three age-subgroups. Scale bar, 10 μm. (**d**) Bone histomorphometry analysis of the age-related changes in BFR/BS at distal femur in three age-subgroups, respectively. (**e**) Correlation analysis between serum exosomal miR-214-3p level and BFR/BS (left), between serum exosomal and intraosteoclast miR-214-3p levels (middle) and between intraosteoclast miR-214-3p level and BFR/BS (right), respectively. The *n* value for each group is indicated at the bottom of each histogram. All data are the mean±s.d. **P<0.01. One-way ANOVA with a *post-hoc* test was performed.

As *miR-214-3p* is evolutionally conserved among several species^23^, we explored the above relationships in an ageing OVX mouse model (Fig. 2a). Consistently, we found an age-related increase in miR-214-3p level in whole serum and serum exosomes from OVX mice, respectively (Fig. 2b). Similarly, the miR-214-3p levels in whole serum and serum exosomes were also almost equal in OVX mice (Supplementary Fig. 2c,d). Bone histomorphometry analysis of distal femur further showed that the bone formation rate per bone surface (BFR/BS) declined with age (Fig. 2c,d). Moreover, the miR-214-3p level in serum exosomes was negatively correlated with the BFR/BS in OVX mice (Fig. 2e).

To determine whether osteoclasts (or osteoblasts) make greater contribution to serum exosomal miR-214-3p rather than osteoblasts (or osteoclasts), we examined the miR-214-3p level in the cultured osteoblasts and osteoclasts, respectively. We found abundant miR-214-3p in mature mouse osteoclasts (mouse OCs) differentiated from bone marrow macrophages (BMMs) but not in mature mouse osteoblasts (mouse OBs) differentiated from calvarial bone-derived osteoblast precursor cells (Supplementary Fig. 3a). Similarly, miR-214-3p was also abundant in the supernatant exosomes of mouse OCs rather than in those of mouse OBs *in vitro* (Supplementary Fig. 3b). We further compared the miR-214-3p level in calvarial bone-derived osteoblast precursor cells/BMMs before and after osteogenic/osteoclastogenic induction, respectively. The mature osteoblasts (ALP^+^ cells) and osteoclasts (OSCAR^+^ cells) were further purified by magnetic-activated cell sorting (MACS). We found that the level of either intracellular miR-214-3p and supernatant exosomal miR-214-3p in mature osteoblasts was lower than that in osteoblast precursors, and the level of intracellular miR-214-3p in ALP^+^ cells (purified osteoblasts) was also lower than that in ALP^−^ cells (non-osteoblasts) (Supplementary Fig. 3c,d). Conversely, the level of either intracellular miR-214-3p or supernatant exosomal miR-214-3p in mature osteoclasts was higher than that in osteoclast precursors, and the level of intracellular miR-214-3p in OSCAR^+^ cells (purified osteoclasts) was also higher than that in OSCAR^−^ cells (non-osteoclasts; Supplementary Fig. 3e,f). Moreover, the level of miR-214-3p was remarkably higher in OSCAR^+^ cells than that in ALP^+^ cells (Supplementary Fig. 3g). Consistently, we found abundant intracellular miR-214-3p and supernatant exosomal miR-214-3p in human osteoclasts (human OCs) differentiated from peripheral blood mononuclear cells (PBMCs) when compared with those in human osteoblasts (human OBs) *in vitro* (Supplementary Fig. 3h,i). The above data suggested that osteoclasts rather than osteoblasts could make greater contribution to the elevated serum exosomal miR-214-3p level. Subsequently, we examined the levels of pri-miR-214-3p, pre-miR-214-3p and mature miR-214-3p in CTSK^+^ cells (osteoclasts) isolated from distal femur cryosections in the above ageing OVX mice by laser-captured microdissection (LCM) in combination with real-time PCR. The purity of osteoclasts isolated by LCM was confirmed by real-time PCR analysis (Supplementary Fig. 4). We found an age-related increase in the level of either pri-miR-214-3p, pre-miR-214-3p or miR-214-3p in osteoclasts, wherein the increased miR-214-3p level was correlated with the increased serum exosomal miR-214-3p level and decreased BFR/BS, respectively (Fig. 2b,e). On the other hand, we found that the levels of pri-miR-214-3p, pre-miR-214-3p and mature miR-214-3p were all remarkably higher in bone marrow–derived OSCAR^+^ cells isolated by MACS when compared with those in OSCAR^−^ cells (Supplementary Fig. 5). Taken together, these data indicate that increased miR-214-3p in osteoclasts associates with reduced bone formation.

### Elevated miR-214-3p in osteoclasts inhibits bone formation

**Figure 3:**
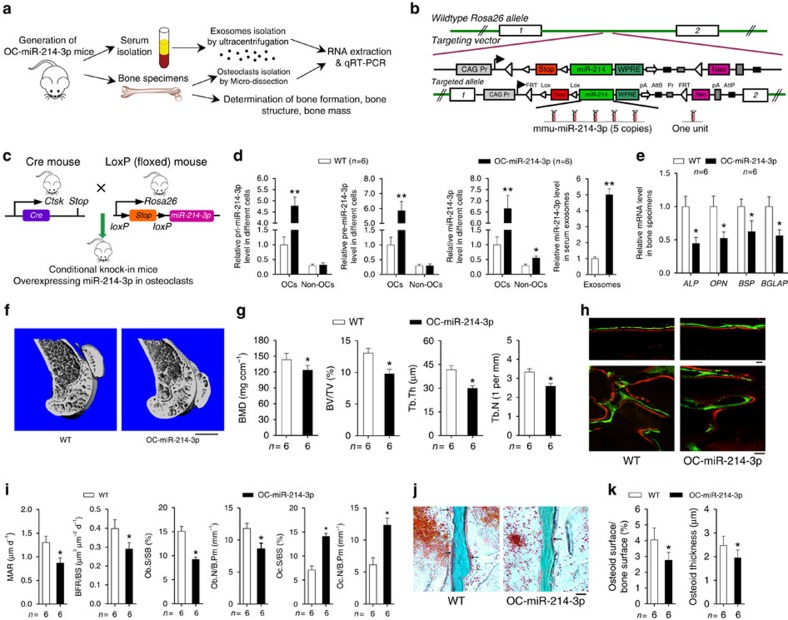
Reduced bone formation in OC-miR-214-3p mice. (**a**) A schematic diagram illustrating the experimental design. (**b**,**c**) Schematic diagrams of the development strategy of the OC-miR-214-3p mice. The ROSA26-miR-214-3p knock-in mice containing the miR-214-3p knock-in allele were generated, and then crossed with the *Ctsk-cre* transgenic mice to obtain the OC-miR-214-3p mice. One unit included the mmu-miR-214-3p stem–loop, a 200-bp 5′ flanking sequence and a 200-bp 3′ flanking sequence. (**d**) Real-time PCR analysis of pri-miR-214-3p (left), pre-miR-214-3p (middle left) and miR-214-3p (middle right) levels in osteoclasts (OCs) versus non-osteoclasts (non-OCs) and serum exosomal miR-214-3p level (right) from WT and OC-miR-214-3p mice. The OCs and non-OCs were the Ctsk^+^ and Ctsk^−^ cells isolated from the bone marrow cells by magnetic-activated cell sorting, respectively. (**e**) Real-time PCR analysis of the mRNA levels of bone formation marker genes (*Alp*, *Opn*, *BSP* and *Bglap*) in bone specimens from WT and OC-miR-214-3p mice. (**f**) Representative micro-CT images of the distal femur from WT and OC-miR-214-3p mice. (**g**) The values of micro-CT parameters (BMD, BV/TV, Tb.Th and Tb.N) at the distal femur metaphysis from WT and OC-miR-214-3p mice. (**h**) Representative images of new bone formation assessed by double labelling with calcein green and xylenol orange at the cortical bone (CB) and trabecular bone (TB) of distal femur from WT and OC-miR-214-3p mice. Scale bars, 10 μm. (**i**) The values of bone histomorphometry parameters (MAR, BFR/BS, Ob.S/BS, Ob.N/B.Pm, Oc.S/BS and Oc.N/B.Pm) at the distal femur metaphysis from WT and OC-miR-214-3p mice. (**j**) Representative images of osteoid formation indicated by Masson's trichrome staining at the distal femur metaphysis of WT and OC-miR-214-3p mice. Scale bars, 20 μm. (**k**) The values of osteoid-related parameters (Osteoid surface/bone surface and Osteoid thickness) at the distal femur metaphysis from WT and OC-miR-214-3p mice. The *n* value for each group is indicated at the bottom of each histogram. All data are the mean±s.d. *P<0.05 versus WT. **P<0.01 versus WT. Student's *t*-test was performed.

To investigate the role of osteoclastic miR-214-3p in regulating bone formation, we generated a mouse strain containing the *miR-214-3p* knock-in allele, then crossed them with the *Ctsk-cre* transgenic mice to obtain the osteoclast-specific *miR-214-3p* knock-in (OC-miR-214-3p) mice (Fig. 3a–c). Then, we examined the bone phenotype in 2-month-old OC-miR-214-3p mice. Real-time PCR analysis showed that the levels of pri-miR-214-3p and pre-miR-214-3p in osteoclasts (CTSK^+^ cells isolated from distal femur cryosections by LCM) and bone tissue were both significantly higher in OC-miR-214-3p mice when compared with those in littermate controls (hereafter wild-type (WT) mice), whereas no significant differences in the levels of pri-miR-214-3p and pre-miR-214-3p in CTSK^−^ cells (non-osteoclasts) and non-skeleton tissues were found between OC-miR-214-3p and WT mice (Fig. 3d and Supplementary Fig. 6). Consistently, the miR-214-3p levels in osteoclasts and serum exosomes were both remarkably higher in OC-miR-214-3p mice than those in WT mice (Fig. 3d). Given that *Ctsk* has been recently reported to be expressed in osteocytes^31^, we examined the miR-214-3p level in osteocytes from either the OC-miR-214 mice or WT mice by Q-PCR analysis. The osteocytic RNA were extracted from tibiae diaphysis after sequential enzymatic digestions^32^. We found no significant difference in the osteocytic miR-214 levels between OC-miR-214 and WT mice (Supplementary Fig. 7). Interestingly, the miR-214-3p level in ALP^+^ cells (osteoblasts) isolated from bone marrow cells by fluorescence-activated cell sorting (FACS) was also significantly higher in OC-miR-214-3p mice when compared with that in WT mice, whereas no obvious difference in the level of either pri-miR-214-3p or pre-miR-214-3p in osteoblasts was found between the OC-miR-214-3p mice and WT mice (Supplementary Fig. 8).

On the other hand, the intra-osseous mRNA levels of bone formation marker genes, including alkaline phosphatase (*Alp*), osteopontin (*Opn*), bone sialoprotein (*Bsp*) and *Bglap*, were all remarkably lower in OC-miR-214-3p mice than those in WT mice (Fig. 3e). Micro-computed tomography (CT) analysis revealed poorly organized trabecular architecture and lower bone mass at distal femur in OC-miR-214-3p mice when compared with those in WT mice (Fig. 3f). Consistently, the micro-CT parameters, including BMD (bone mineral density), BV/TV (bone volume/total volume), Tb.Th (trabecular thickness) and Tb.N (trabecular numbers), were all significantly lower in OC-miR-214-3p mice than those in WT mice (Fig. 3g). Qualitatively, undecalcified bone histology showed that the width between double labelling at distal femur was smaller in OC-miR-214-3p mice than that in WT mice (Fig. 3h). Quantitatively, bone histomorphometry analysis of distal femur showed that the MAR (mineral apposition rate), BFR/BS, Ob.S/BS (the percent of trabecular bone surface covered by osteoblasts) and Ob.N/B.Pm (osteoblast number per bone perimeter) were all significantly lower in OC-miR-214-3p mice when compared with those in WT mice (Fig. 3i). In addition, masson's trichrome staining of the undecalcified bone section also showed less osteoid staining at distal femur in OC-miR-214-3p mice as compared with that in WT mice (Fig. 3j,k). All these data suggest that elevated miR-214-3p in osteoclasts could result in reduced bone formation. Interestingly, we also found that the Oc.S/BS (the percent of trabecular bone surface covered by osteoclasts,) and Oc.N/B.Pm (osteoclast number per bone perimeter) at distal femur were both remarkably higher in OC-miR-214-3p mice when compared with those in WT mice (Fig. 3i), and miR-214-3p could promote osteoclast differentiation *in vitro* (Supplementary Fig. 9), suggesting a dual role of miR-214-3p in regulating bone formation and bone resorption.

**Figure 4:**
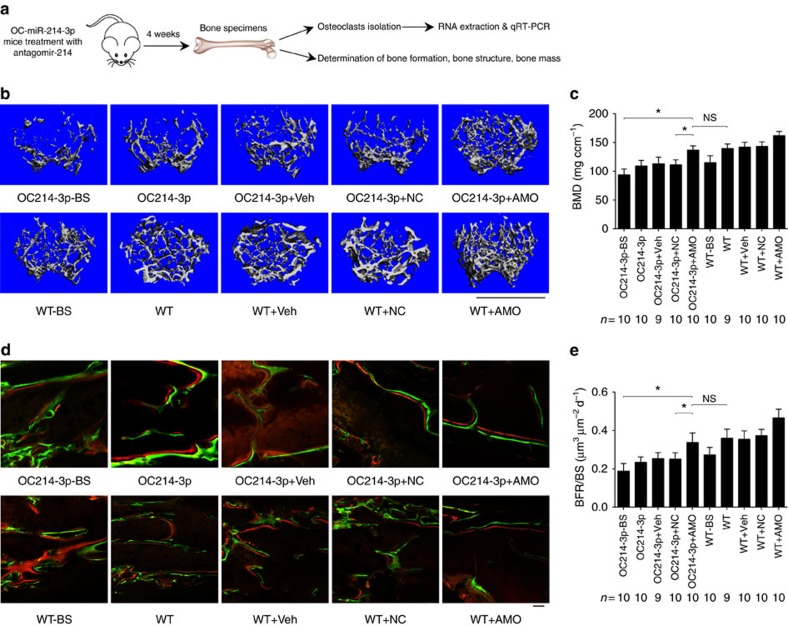
Osteoclast-targeted antagomir-214-3p treatment rescues bone phenotype in OC-miR-214-3p mice. (**a**) A schematic diagram illustrating the experimental design. The 4-week-old OC-miR-214-3p or WT mice were intravenously injected with PBS (OC214-3p/WT), (D-Asp)_8_-liposome (vehicle) alone (OC214-3p+Veh/WT+Veh), (D-Asp)_8_-liposome-antagomir nonsense control (OC214-3p+NC/WT+NC) and (D-Asp)_8_-liposome-antagomir-214-3p (OC214-3p+AMO/WT+AMO), respectively, at a weekly interval and killed 4 weeks after the first treatment. Another group of OC-miR-214-3p or WT mice were killed at 4-week-old before treatment initiation as baseline (OC214-3p-BS/WT-BS). (**b**) Representative micro-CT images of the distal femur metaphysis in each group. Scale bars, 1 mm. (**c**) The values of micro-CT parameter (BMD) at the distal femur metaphysis in each group. (**d**) Representative images of new bone formation assessed by double labelling with calcein green and xylenol orange at the distal femur metaphysis in each group. Scale bars, 10 μm. (**e**) The values of bone histomorphometry parameter (BFR/BS) at the distal femur metaphysis in each group. The *n* value for each group is indicated at the bottom of each histogram. All data are the mean±s.d. *P<0.05. NS, not significant. One-way ANOVA with a *post-hoc* test was performed.

To examine whether the bone phenotype of OC-miR-214-3p mice could be rescued by therapeutic inhibition of miR-214-3p in osteoclasts, we performed weekly pulsed-injections of antagomiR-214-3p (AMO, 10 mg kg^−1^) encapsulated by our previously developed osteoclast-targeting delivery system, that is, (D-Asp_8_)-liposome^33^, in 4-week-old OC-miR-214-3p mice (Fig. 4a). Immunohistochemistry and real-time PCR analysis at 24 h after AMO injection confirmed that antagomir-214-3p could be effectively delivered to osteoclasts *in vivo*, as evidenced by the numerous instances of co-localization of fluorescein amidite (FAM)-labelled antagomiR-214-3p with CTSK^+^ cells (osteoclasts) in distal femur cryosections and the downregulated miR-214-3p level in CTSK^+^ cells isolated by LCM (Supplementary Fig. 10). Four weeks after the first AMO injection, micro-CT analysis revealed significantly higher bone mass with well-organized trabecular architecture at distal femur in OC-miR-214-3p+AMO mice, which was similar to those in WT mice (Fig. 4b). Consistently, the values of micro-CT parameters in OC-miR-214-3p+AMO mice were almost restored to the levels in WT mice (Fig. 4c and Supplementary Fig. 11a). The undecalcified bone histology showed that the width between xylenol orange and calcein green labelling at distal femur in OC-miR-214-3p+AMO mice resembled that in WT mice (Fig. 4d). Bone histomorphometric analysis also showed that the bone formation-related parameters (MAR, BFR/BS, Ob.S/BS and Ob.N/B.Pm) and bone resorption-related parameters (Oc.S/BS and Oc.N/B.Pm) in OC-miR-214-3p+AMO mice were almost restored to the levels in WT mice, respectively (Fig. 4e and Supplementary Fig. 11b). Taken together, these data suggest that elevated miR-214-3p in osteoclasts could inhibit bone formation.

### Exosomal miR-214-3p from osteoclasts inhibits osteoblast activity

**Figure 5:**
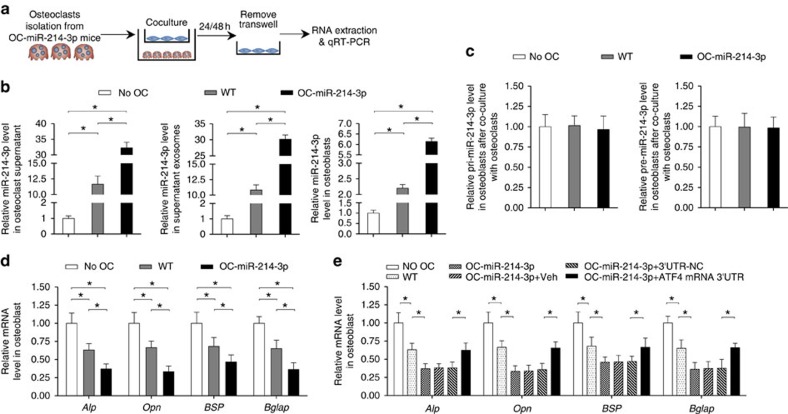
Osteoclastic miR-214-3p inhibit osteoblast activity. (**a**) A schematic diagram illustrating the design of the co-culture experiments. The osteoblasts derived from the calvarial bone of newborn C57BL/6 mice were co-cultured with the osteoclasts derived from OC-miR-214-3p mice (OC-miR-214-3p OCs) or WT mice (WT OCs), or cultured without osteoclasts (No OC). (**b**) Real-time PCR analysis of the supernatant, supernatant exosomal and intra-osteoblast miR-214-3p levels (normalized by the mean value of No OC) at 24 h after co-culture, respectively. (**c**) The pri-miR-214-3p and pre-miR-214-3p levels in osteoblasts at 24 h after co-culture. (**d**) Real-time PCR analysis of the mRNA levels of osteoblast activity-related marker genes (*Alp*, *Opn*, *BSP* and *Bglap*) in osteoblasts at 48 h after co-culture. (**e**) Real-time PCR analysis of the mRNA expression levels of *Alp*, *Opn*, *BSP* and *Bglap* in osteoblasts transfected with exogenous ATF4 mRNA 3′UTR at 48 h after co-culture. The osteoblasts transfected with either vehicle (OC-miR-214 3p+Veh), nonsense 3′UTR control (OC-miR-214 3p+NC) or ATF4 mRNA 3′UTR (OC-miR-214 3p+ ATF4 mRNA 3′UTR) were co-cultured with the OC-miR-214 3p osteoclasts. All data are the mean±s.d. of four independent experiments. *P<0.05. One-way ANOVA with a *post-hoc* test was performed.

To delineate whether exosomal miR-214-3p transferred from osteoclasts could inhibit osteoblast activity, we co-cultured osteoclasts with osteoblasts in a Transwell system with a 0.4-μm pore polyethylene terephthalate (PET) membrane that allowed transfer of exosomes (50–150 nm) but blocked most of the other shed microvesicles (0.4–1 μm in diameter) and apoptotic bodies (diameter>1 μm; ref. 34; Fig. 5a). The osteoclasts were differentiated from BMMs from the OC-miR-214-3p and WT mice, respectively, under the stimulation of macrophage colony-stimulating factor (M-CSF) and receptor activator for nuclear factor-κB ligand (RANKL)^35^. The osteoblasts were differentiated from the primary osteoblast precursor cells isolated from the calvarial bone of newborn mice in osteogenic medium^36^. Thereafter, the osteoblasts were co-cultured with OC-miR-214-3p and WT osteoclasts, respectively, wherein the supernatant were harvested for exosomes isolation and miRNAs extraction. Western blot analysis of the exosome markers CD9, CD63 and TSG101 in the extracts confirmed the presence of exosomes (Supplementary Fig. 12a). To exclude the possibility that the elevated supernatant miR-214-3p level was caused by the increased exosome production in osteoclasts, the number of exosomes was assessed by nanosight technology. However, no difference in the number of supernatant particles was detected between the two co-cultured system after normalization with the protein content of cell lysates (Supplementary Fig. 12b).

Real-time PCR analysis revealed that the miR-214-3p level in either supernatant or supernatant exosomes in the co-culture of osteoblasts and OC-miR-214-3p osteoclasts was dramatically higher than those in the co-culture of osteoblasts and WT osteoclasts at 24 h after co-culture (Fig. 5b). Consistently, the miR-214-3p level in osteoblasts co-cultured with OC-miR-214-3p osteoclasts was significantly upregulated as compared with that in osteoblasts co-cultured with WT osteoclasts (Fig. 5b). However, no significant difference in the level of either pri-miR-214-3p or pre-miR-214-3p in osteoblasts was found between the two co-cultures (Fig. 5c). Thereafter, we examined the mRNA expression of osteoblast activity-related marker genes (*Alp*, *Opn*, *Bsp* and *Bglap*) in osteoblasts at 48 h after co-culture with OC-miR-214-3p and WT osteoclasts, respectively. The mRNA levels of those genes were all remarkably downregulated in osteoblasts co-cultured with OC-miR-214-3p osteoclasts when compared with those in osteoblasts co-cultured with WT osteoclasts (Fig. 5d). These results indicated that elevated miR-214-3p in osteoclasts could contribute to the upregulated miR-214-3p in osteoblasts and the downregulated osteoblast activity *in vitro*. As we have previously demonstrated that miR-214-3p directly targets the 3′-untranslated region (UTR) of ATF4 mRNA to inhibit osteoblast activity^23^, to further verify whether the downregulated osteoblast activity was caused by the miR-214-3p in the co-cultured osteoclasts, we transfected osteoblasts with lentiviral vector for expression of exogenous ATF4 mRNA 3′UTR (LV-ATF4 3′UTR) before co-culture with OC-miR-214-3p osteoclasts. We postulated that the transfected ATF4 mRNA 3′UTR could compete with the endogenous ATF4 mRNA to combine with the transferred miR-214-3p from OC-miR-214-3p osteoclasts. As expected, the mRNA levels of osteoblast activity-related marker genes in the osteoblasts transfected with LV-ATF4 3′UTR and co-cultured with OC-miR-214-3p osteoclasts were partially restored to the levels in the non-transfected osteoblasts co-cultured with WT osteoclasts (Fig. 5e). These data hinted that elevated miR-214-3p in osteoclasts could inhibit osteoblast activity *in vitro*.

**Figure 6:**
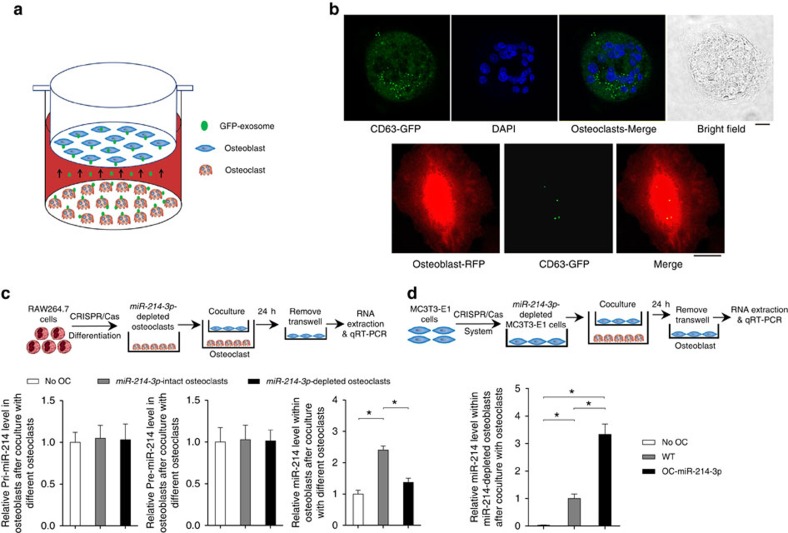
Osteoclast-derived exosomal miR-214-3p transfer to osteoblasts. (**a**) A schematic diagram illustrating the design of co-culture experiments with GFP-exosome-producing osteoclasts and osteoblasts. CMV-GFP-CD63 were transfected into the OC-miR-214-3p osteoclasts to label the osteoclast-derived exosomes. (**b**) Representative confocal images of the GFP-exosome-producing osteoclasts at 24 h after transfection (left panels), and the uptake of GFP-exosome by osteoblasts (right panels) at 24 h after co-culture. (**c**) A schematic diagram illustrating the design of co-culture experiments with the osteoblasts and *miR-214-3p*-depleted osteoclasts (upper panels), and real-time PCR analysis of the levels of pri-miR-214-3p, pre-miR-214-3p and miR-214-3p in osteoblasts (normalized by the mean value of No OC group) at 24 h after co-culture with *miR-214-3p*-depleted/intact osteoclasts differentiated from *miR-214-3p*-depleted/intact RAW264.7 cells, respectively (lower panels). (**d**) A schematic diagram illustrating the design of co-culture experiments with the osteoclasts and *miR-214-3p*-depleted osteoblasts (upper panels), and real-time PCR analysis of the miR-214-3p level in *miR-214-3p*-depleted osteoblasts (normalized by the mean value of No OC group) at 24 h after co-culture with either OC-miR-214-3p or WT osteoclasts (lower panels). All data are the mean±s.d. of four independent experiments. *P<0.05. One-way ANOVA with a *post-hoc* test was performed.

Given that exosomes could protect miRNAs from RNase-induced degradation and mediate intercellular communication^37^, we next verifed whether exosomal miR-214-3p was transferred from osteoclasts to osteoblasts. We constructed a lentivector system of cytomegalovirus (CMV)-driven green fluorescence protein (GFP)-tagged CD63 (CMV-GFP-CD63)^34^ to label the exosomes derived from osteoclasts with GFP. The OC-miR-214-3p osteoclasts were transfected with CMV-GFP-CD63 before co-culture with osteoblasts (Fig. 6a). We observed numerous GFP^+^ particles within osteoblasts after 24 h co-culture with OC-miR-214-3p osteoclasts by confocal imaging (Fig. 6b). To confirm whether osteoclast-derived exosomal miR-214-3p contributes to the elevated miR-214-3p in osteoblasts, the miR-214-3p-depleted osteoclasts and the *miR-214-3p*-depleted osteoblasts were differentiated from the *miR-214-3p-*depleted RAW264.7 cell line and the *miR-214-3p*-depleted MC3T3-E1 cell line, respectively, wherein *miR-214-3p* gene was depleted using the CRISPR-Cas9 system^38^ (Supplementary Fig. 13). We found that the miR-214-3p level in osteoblasts co-cultured with *miR-214-3p*-depleted osteoclasts was significantly lower than that in osteoblasts co-cultured with *miR-214-3p*-intact osteoclasts, whereas no significant difference in the level of either pri-miR-214-3p or pre-miR-214-3p level was found between the osteoblasts co-cultured with miR-214-3p-depleted osteoclasts and miR-214-3p-intact osteoclasts (Fig. 6c). On the other hand, the miR-214-3p level in miR-214-3p-depleted osteoblasts co-cultured with OC-miR-214-3p osteoclasts was significantly higher than that in miR-214-3p-depleted osteoblasts co-cultured with WT osteoclasts (Fig. 6d). Taken together, these results indicate that exosomal miR-214-3p could be transferred from osteoclasts to osteoblasts to inhibit osteoblast activity *in vitro*.

### Osteoclast-derived exosomal miR-214-3p inhibits bone formation

**Figure 7:**
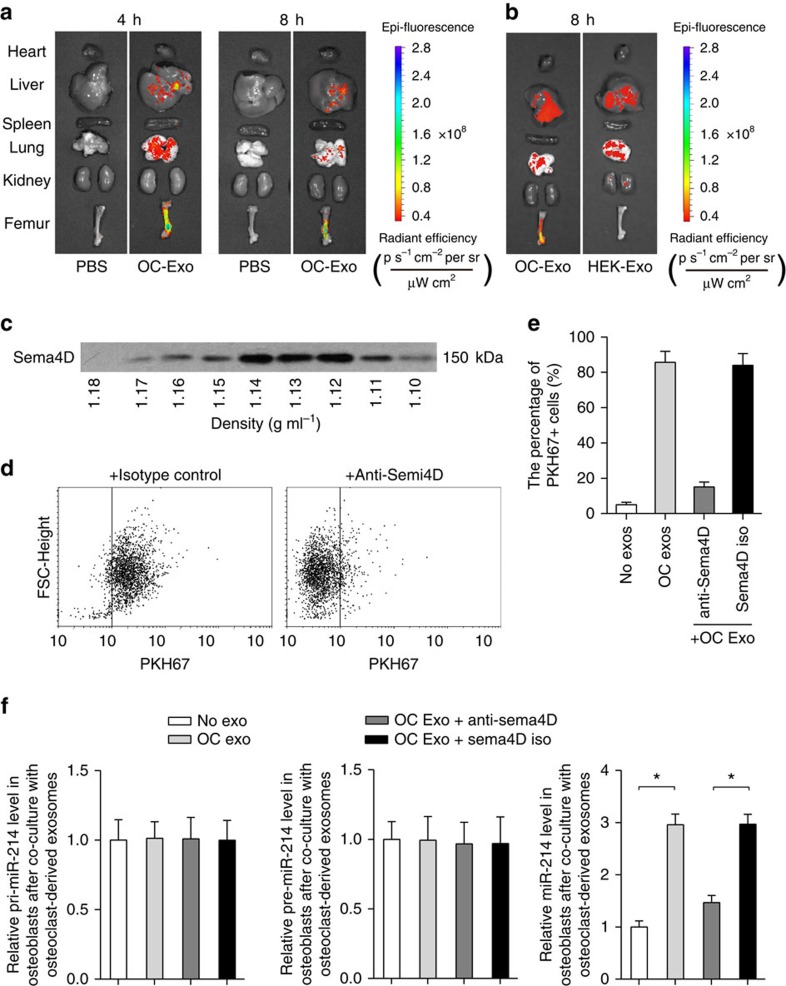
Osteoclast-derived exosomes target osteoblasts. (**a**) Representative biophotonic images of the tissue distribution of fluorescence signal in mice at 4 and 8 h after intravenous injection of purified PKH67-labelled exosomes isolated from the supernatant of OC-miR-214-3p osteoclasts (OC-Exo). (**b**) Representative biophotonic images of the tissue distribution of fluorescence signal in mice at 8 h after intravenous injection of purified PKH67 exosomes isolated from the supernatant of either OC-miR-214-3p osteoclast (OC-Exo) or HEK 293T cells (HEK-Exo). (**c**) Western blot analysis of the Sema4D protein expression in pellets of sucrose gradient fractions from the osteoclast-derived exosome preparations. The density of each fraction was determined by refraction index measurements. (**d**,**e**) Flow cytometry analysis of PKH67^+^ osteoblasts after incubation with PKH67-labelled exosomes derived from OC-miR-214-3p osteoclasts. The PKH67-labelled exosomes were pre-treated with either anti-Sema4D (20 μg ml^−1^) or isotype-matched control (Sema4D iso) and then added to osteoblasts for 4 h incubation. (**f**) Real-time PCR analysis of the levels of pri-miR-214, pre-miR-214 and mature miR-214 in osteoblasts after incubation with either anti-Sema4D-treated exosomes (OC Exo+Anti-Sema4D) or Sema4D isotype-treated exosomes (OC Exo+Sema4D iso), respectively. All data are the mean±s.d. of four independent experiments. *P<0.05. One-way ANOVA with a *post-hoc* test was performed.

To examine whether exosomal miR-214-3p derived from osteoclasts could inhibit bone formation *in vivo*, a batch of 3-month-old female C57BL/6J mice were intravenously injected with PKH67-labelled exosomes (100 μg per mouse) isolated and purified from the supernatant of OC-miR-214-3p osteoclasts or equal volume of phosphate-buffered solution (PBS, set as negative control). The distribution of PKH67-exosomes was evaluated by biophotonic imaging at 4 and 8 h after injections, respectively. The intra-osseous fluorescence signal was detected in mice administrated with PKH67-exosomes at either 4 or 8 h after administration, whereas it was not detectable in mice administrated with PBS at each time point (Fig. 7a). Thereafter, we performed immunohistochemistry analysis to investigate the uptake of PKH67 exosomes in osteoblasts *in vivo* at 8 h after injection. We found some instances of co-localization of PKH67-positive particles with ALP^+^ cells (osteoblasts) in the cryosections of distal femur in mice administrated with PKH67 exosomes, whereas the florescence signal was absent in mice treated with PBS (Supplementary Fig. 14). To address if there was real bone-target specificity of the osteoclast-derived exosomes, we treated another batch of female C57BL/6 mice with PKH67-labelled exosomes isolated and purified from the supernatant of either osteoclasts or HEK 293T cells. Biophotonic imaging showed that the intraosseous fluorescence signal was detected in mice administrated with PKH67-exosomes derived from osteoclasts but not in mice administrated with PKH67 exosomes derived from HEK 293T cells at 8 h after administration (Fig. 7b). In addition, the Sema4D, a previously identified osteoclast membrane protein that could target its receptor (Plexin B) in osteoblasts^6^, was also detected in osteoclast-derived exosomes by western blot analysis (Fig. 7c). Accordingly, the interaction between osteoclast-derived exosomes and osteoblasts was interrupted *in vitro* after treatment of Sema4D antibody (Fig. 7e,f).

**Figure 8:**
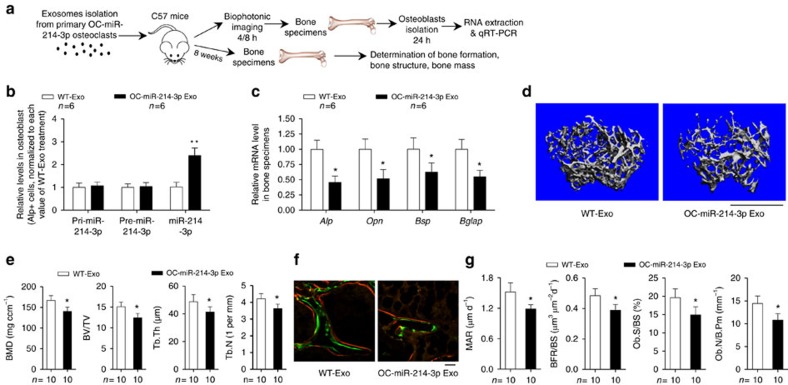
Osteoclast-derived exosomal miR-214-3p inhibit bone formation. (**a**) A schematic diagram illustrating the experimental design. The female C57BL/6 mice were intravenously injected with purified exosomes derived from either OC-miR-214-3p (OC-miR-214-3p Exo) or WT (WT-Exo) osteoclasts. (**b**) Real-time PCR analysis of the levels of either pri-miR-214, pre-miR-214 or miR-214 in ALP^+^ cells (osteoblasts) isolated from bone marrow cells by fluorescence-activated cell sorting at 24 h after the mice were intravenously injected with either OC-miR-214-3p Exo or WT-Exo. (**c**) Real-time PCR analysis of the mRNA expression levels of *Alp*, *Opn*, *BSP* and *Bglap* in femurs from the mice administered with either OC-miR-214-3p Exo or WT-Exo, respectively. (**d**) Representative micro-CT images of the distal femur metaphysis from the mice administered with either OC-miR-214-3p Exo or WT-Exo. Scale bar, 1 mm. (**e**) The values of micro-CT parameters (BMD, BV/TV, Tb.Th and Tb.N) at the distal femur metaphysis from the mice administered with either OC-miR-214-3p Exo or WT-Exo, respectively. (**f**) Representative images of new bone formation assessed by double labelling with calcein green and xylenol orange at the distal femur metaphysis from the mice administered with either OC-miR-214-3p Exo or WT-Exo. Scale bars, 10 μm. (**g**) The values of bone histomorphometry parameters (MAR, BFR/BS, Ob.S/BS, Ob.N/B.Pm) at the distal femur metaphysis from the mice administered with either OC-miR-214-3p Exo or WT-Exo, respectively. The *n* value for each group is indicated at the top/bottom of each histogram. All data are the mean±s.d. *P<0.05 versus WT. Student's *t*-test was performed.

Subsequently, another batch of 3-month-old female C57BL/6J mice were intravenously injected with exosomes (100 μg per mouse) isolated and purified from the supernatant of either OC-miR-214-3p osteoclasts (OC-miR-214-3p-exosomes) or WT osteoclasts (WT-exosomes; Fig. 8a). Then, we performed real-time PCR to examine the miR-214-3p level in osteoblasts (ALP^+^ cells) isolated from bone marrow cells by FACS at 24 h after exosome treatments. As expected, the miR-214-3p level in osteoblasts was significantly higher in mice administrated with OC-miR-214-3p-exosomes when compared with that in mice treated with WT exosomes, whereas no change of the level of either pri-miR-214-3p or pre-miR-214-3p in osteoblasts was found between OC-miR-214-3p exosomes treatment and WT exosomes treatment (Fig. 8b). Thereafter, we investigated the effect of OC-miR-214-3p exosomes on osteoblastic bone formation *in vivo*. Another batch of 3-month-old female C57BL/6J mice received eight consecutive intravenous injections of either OC-miR-214-3p exosomes or WT exosomes (100 μg per mouse) at a weekly interval and killed 1 week after the last injection (Fig. 8a). Real-time PCR analysis of distal femur showed that the intra-osseous mRNA levels of bone formation marker genes *Alp, Opn, Bsp* and *Bglap* were remarkably lower in mice treated with OC-miR-214-3p exosomes when compared with those in mice treated with WT exosomes (Fig. 8c). Micro-CT analysis showed reduced bone mass (lower BMD and BV/TV) as well as poorly organized trabecular architecture (lower Tb.Th and Tb.N) at distal femur in mice treated with OC-miR-214-3p exosomes (Fig. 8d,e). Bone histomorphometric analysis showed that the MAR, BFR/BS, Ob.S/BS and Ob.N/B.Pm at distal femur were all lower in mice treated with OC-miR-214-3p exosomes than those in mice treated with WT exosomes (Fig. 8f,g). Taken together, these data imply that exosomal miR-214-3p derived from osteoclasts could inhibit bone formation *in vivo*.

### Inhibition of miR-214-3p in osteoclasts promotes bone formation

**Figure 9:**
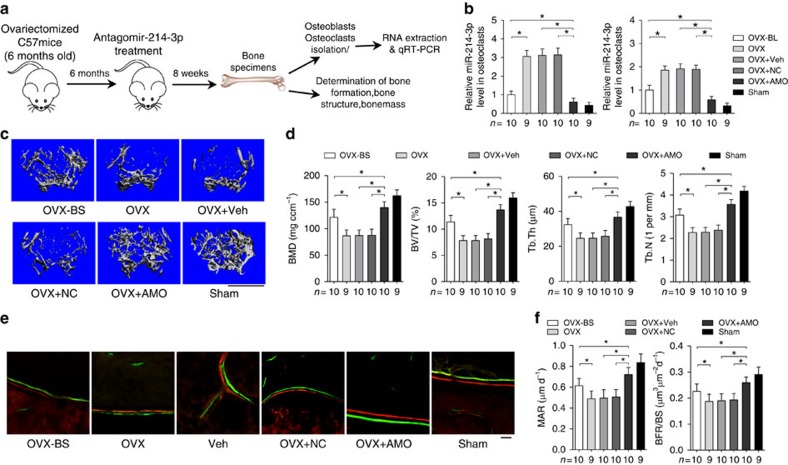
Osteoclast-targeted antagomiR-214-3p treatment promotes bone formation in ageing OVX mice. (**a**) A schematic diagram illustrating the experimental design. The female C57BL/6 mice were either ovariectomized or Sham operated at 6-month-old, and left untreated for 6 months. At 12-month-old, the OVX mice received eight consecutive intravenous injections with either PBS (OVX), (D-Asp)8-liposome (vehicle) alone (OVX+Veh), (D-Asp)8-liposome-antagomir nonsense control (OVX+NC) or (D-Asp)8-liposome-antagomir-214-3p (OVX+AMO), whereas the Sham mice received eight consecutive intravenous injections with PBS (Sham), at a weekly interval and killed 8 weeks after the first injection. Another group of OVX mice were killed at 12-month-old before treatment initiation as baseline (OVX-BS). (**b**) Real–time PCR analysis of the miR-214-3p levels in CTSK^+^ cells (Osteoclasts) and ALP^+^ cells (osteoblasts) isolated from cryosections of femur by laser-captured microdissection from the mice in each group. (**c**) Representative micro-CT images of the distal femur metaphysis from the mice in each group. Scale bars, 1 mm. (**d**) The values of micro-CT parameters (BMD, BV/TV, Tb.Th and Tb.N) at the distal femur metaphysis from the mice in each group. (**e**) Representative images of new bone formation assessed by double labelling with calcein green and xylenol orange at the distal femur metaphysis from the mice in each group. Scale bars, 10 μm. (**f**) The values of bone histomorphometry parameters (MAR, BFR/BS) at the distal femur metaphysis from the mice in each group. The *n* value for each group is indicated at the top/bottom of each histogram. All data are the mean±s.d. *P<0.05. One-way ANOVA with a *post-hoc* test was performed.

To test whether miR-214-3p inhibition in osteoclasts could promote bone formation, we performed pulsed administration of antagomir-214-3p (AMO) encapsulated by the aforementioned osteoclast-targeting delivery system^33^ in an ageing OVX mouse model. Six-month-old female C57BL/6J mice were OVX, left untreated for 6 months followed by eight consecutive intravenous injections of AMO (10 mg kg^−1^) encapsulated by (D-Asp_8_)-liposome at a weekly interval (Fig. 9a). Eight weeks after the first injection, we performed real-time PCR analysis to examine the miR-214-3p level in osteoclasts (CTSK^+^ cells) and osteoblasts (ALP^+^ cells) isolated from distal femur cryosections by LCM, respectively. We found that the miR-214-3p levels in osteoclasts and osteoblasts were both remarkably lower in OVX mice treated with antagomir-214-3p (OVX+AMO), but significantly higher in OVX mice treated with either PBS (OVX), vehicle (OVX+Veh) or antagomir-negative control (OVX+NC), when compared with those in mice at baseline (Fig. 9b).

As revealed by micro-CT and bone histomorphometry analysis, the sham-operated mice had substantially higher trabecular bone mass and better trabecular architecture as well as higher MAR and BFR/BS at distal femur when compared with those in OVX mice (Fig. 9c–f). Moreover, micro-CT analysis of distal femur showed that the trabecular bone mass was notably reduced (lower in both BMD and BV/TV) and the trabecular architecture was dramatically impaired (lower in both Tb.Th and Tb.N) in OVX, OVX+Veh and OVX+NC mice, respectively, when compared with OVX mice at baseline, whereas the trabecular bone mass was remarkably higher and the trabecular architecture was markedly improved in OVX+AMO mice (Fig. 9c,d). Accordingly, bone histomorphometry analysis of distal femur showed that the bone-formation-related parameters (MAR, BFR/BS, Ob.S/BS and Ob.N/B.Pm) were notably lower, and the bone resorption-related parameters (Oc.S/BS and Oc.N/B.Pm) were slightly higher in either OVX, OVX+Veh or OVX+NC mice when compared with OVX mice at baseline (Fig. 9e,f and Supplementary Fig. 15). However, the bone formation-related parameters were remarkably higher and the bone resorption-related parameters were significantly lower in OVX+AMO mice when compared with OVX mice at baseline (Fig. 9e,f and Supplementary Fig. 15).

To confirm that the above therapeutic effect was attributed to the osteoclast-targeted-delivery of antagomir-214-3p but not to the uptake of antagomir-214-3p in other bone marrow cells that could be potential sources of exosomal miR-214-3p, we investigated whether blocking the interaction between the targeting moiety (D-Asp_8_) and bone resorption surfaces by pretreatment with D-Asp_8_ could abolish the beneficial effect of osteoclast-targeted-delivery of antagomir-214-3p on bone formation. Impressively, the result showed that the reducing effect of (D-Asp_8_)-lipsome-antagomir-214-3p treatment on miR-214-3p levels in osteoclasts and osteoblasts were almost prevented in those OVX mice pretreated with D-Asp_8_ 24 h ahead of each AMO treatment (Supplementary Fig. 16a). Consistently, the beneficial effect on bone structure and bone formation in OVX mice by (D-Asp_8_)-lipsome-antagomir-214-3p treatment were disappeared in those OVX mice pretreated with D-Asp_8_ 24 h ahead of each AMO treatment (Supplementary Fig. 16b–e). Collectively, these results indicate that osteoclast-targeted inhibition miR-214-3p could promote bone formation in ageing OVX mice.

## Discussion

In this study, we performed a series of *in vitro* and *in vivo* studies to identify that osteoclast-derived exosomal miR-214-3p could transfer to osteoblasts to inhibit bone formation, proposing a paradigm of miRNA-mediated osteoclast-to-osteoblast communication for participation in homeostasis mechanism of local bone environment.

We found a close association between the elevated miR-214-3p in osteoclasts and reduced bone formation, as evidenced by the negative correlation between the intra-osseous miR-214-3p level and *BGLAP* mRNA level in elderly women with fractures, and between the intra-osteoclast miR-214-3p level and bone formation rate in OVX mice. To delineate the role of osteoclastic miR-214-3p in regulating bone formation, we generated a genetic mouse model in which miR-214-3p was specifically overexpressed in osteoclasts (OC-miR-214-3p mice). It has been reported that miR-214-3p promotes osteoclast differentiation by targeting the Pten/PI3k/Akt pathway^24^. Consistently, we observed the elevated bone resorption in OC-miR-214-3p mice and found that miR-214-3p could promote osteoclast differentiation *in vitro*. More importantly, we found that elevated miR-214-3p in osteoclasts could result in reduced bone formation, which could be rescued by osteoclast-targeted inhibition of miR-214-3p. In addition, the *in vitro* data further illustrated that elevated miR-214-3p in osteoclast could suppress osteoblast activity, as evidenced by the markedly downregulated mRNA levels of osteoblast activity-related marker genes in the osteoblasts co-cultured with the osteoclasts overexpressing miR-214-3p. Thus, all these data indicate that the aberrantly elevated miR-214-3p in osteoclasts may contribute to the suppression on osteoblast activity and reduction on bone formation.

In addition, we found a positive correlation between the serum exosomal miR-214-3p level and intra-osseous miR-214-3p level in elderly women with fractures and between the serum exosomal miR-214-3p level and intra-osteoclast miR-214-3p level in OVX mice, respectively. Similarly, the serum exosomal miR-214-3p level was also significantly higher in OC-miR-214-3p mice compared with WT controls. Furthermore, the osteoclast marker proteins (CTSK, TRAcP5 and Sema4D) were detectable in serum exosomes isolated from elderly individuals with or without fractures. In addition, our *in vitro* data postulated that the osteoclasts rather than osteoblasts could contribute to the serum exosomal miR-214-3p, as evidenced by the abundant miR-214-3p in supernatant exosomes of the osteoclasts (mouse and human OCs) rather than the osteoblasts (mouse and human OBs) *in vitro*. Thus, it hints that miR-214-3p in osteoclasts could be released in exosome-encapsulated form. However, because of the technical limitations, we could not distinguish the osteoclast-derived exosomes from other serum vesicles that released by non-osteoclasts. Therefore, at this stage, it could not be excluded that exosomes derived from non-osteoclasts, for example, endothelial cells-derived exosomes^39^, may also contribute to the serum exosomal miR-214-3p level. Interestingly, we found elevated miR-214-3p in serum exosome as well as increased mature miR-214-3p with no changes of pri-miR-214-3p and pre-miR-214-3p in osteoblasts of OC-miR-214-3p mice compared with WT mice, suggesting that osteoclastic miR-214-3p could contribute to the miR-214-3p in serum exosome and osteoblasts.

Thereafter, we performed a series of studies to rigorously address whether osteoclast-derived exosomal miR-214-3p released from could be transferred into osteoblasts to inhibit bone formation. First, to investigate whether osteoclastic miR-214-3p could transferred into osteoblasts, we co-cultured osteoblasts with either OC-miR-214-3p or WT osteoclasts, and found that the level of mature miR-214-3p was higher, whereas the level of either pri-miR-214-3p or pre-miR-214-3p was unchanged, in osteoblasts co-cultured with OC-miR-214-3p osteoclasts when compared with those in osteoblasts co-cultured with WT osteoclasts. Similarly, the level of mature miR-214-3p also significantly increased in *miR-214-3p*-depleted osteoblasts after co-culture with the OC-miR-214-3p osteoclasts, whereas the level of either mature miR-214-3p, pri-miR-214-3p or pre-miR-214-3p did not significantly increase in osteoblasts after co-culture with *miR-214-3p-*depleted osteoclasts. These results suggest that the exogenous osteoclastic miR-214-3p could transfer to become constitutional part of mature miR-214-3p in osteoblasts. Next, we performed *in vitro* and *in vivo* exosome-tracking experiments to investigate whether osteoclastic exosomal miR-214-3p could be transferred into osteoblasts. We transfected the OC-miR-214-3p osteoclasts with CMV-GFP-CD63 to label the osteoclast-derived exosomes and detected numerous GFP-positive particles in the co-cultured osteoblasts. Consistently, after the mice were injected with PKH67-labelled exosomes derived from OC-miR-214-3p osteoclasts, we detected intra-osseous fluorescence signal and observed co-localization of PKH67-positive particles with osteoblasts *in vivo*. However, no intra-osseous fluorescence signal was detected in mice administrated with PKH67 exosomes derived from HEK 293T cells. Furthermore, we found increased miR-214-3p level with no changes in the level of either pri-miR-214-3p or pre-miR-214-3p in osteoblasts after injecting the above osteoclast-derived exosomes *in vivo*. In addition, although there is no established method to specifically block the exosome-mediated osteoclast-to-osteoblast communication *in vivo*, we found that blocking Sema4D in osteoclast-derived exosomes could interrupt the interaction between osteoclast-derived exosomes and osteoblasts *in vitro*. All these data suggest that exosomal miR-214-3p could be transferred from osteoclasts to osteoblasts. To investigate whether the osteoclast-derived exosomal miR-214-3p could be transferred into osteoblasts to inhibit bone formation, we transfected the osteoblasts with ATF4 3′UTR and found that the repressed osteoblast activity after co-culture with OC-miR-214-3p osteoclasts could be remarkably restored. More importantly, the *in vivo* data showed that administration of exosomes derived from the OC-miR-214-3p osteoclasts could result in reduced bone formation. Collectively, our results indicate that osteoclast-derived exosomal miR-214-3p could be transferred into osteoblasts to inhibit osteoblastic bone formation.

Besides the in-depth studies on the regulation of osteoclast function and osteoclastic bone resorption by osteoblast-derived molecules^40^, a number of recent studies have uncovered the important role of osteoclast-dependent osteoblastic bone formation^13^,^14^. Moreover, a novel function of miRNAs as the intercellular signal was also demonstrated in several recent studies^17^,^18^,^19^,^20^,^39^. On the other hand, the intercellular transportation of miRNAs was considered to be mediated by extracellular vesicles including exosome, which pinches off from the plasma membrance and represent a population of membrane vesicles that could protect miRNAs from RNase-induced degradation^37^,^41^. Thus, it would be reasonable that osteoclasts could communicate with osteoblasts using miRNA signal. In the present study, we identified that exosomal miR-214-3p could be transferred from osteoclasts to osteoblasts to regulate bone formation, serving as an intercellular messenger to mediate osteoclast-to-osteoblast communication.

Considering the role of exosomal miR-214-3p derived from osteoclasts in regulating osteoblastic bone formation, inhibition of this miRNA in osteoclasts may exert beneficial effect on bone formation. Thus, we evaluated the therapeutic effect of osteoclast-targeted miR-214-3p inhibition by administration of antagomiR-214-3p delivered by our recently developed osteoclast-targeting delivery system^33^. We found that antagomir-214-3p treatment significantly promoted bone formation in ageing OVX mice, while such beneficial effect was blocked after the osteoclast-targeting delivery mechanism was interrupted. All these results suggest that inhibition of miR-214-3p in osteoclasts could promote bone formation and increase bone mass in ageing OVX mice.

Taken together, our study suggests that exosomal miR-214-3p could serve as an intercellular messenger to mediate osteoclast-to-osteoblast communication for inhibiting osteoblastic bone formation. Towards translational medicine, therapeutic inhibition of miR-214-3p in osteoclasts may be a potential bone anabolic strategy to reverse the established osteoporosis.

## Methods

### Human serum and bone specimen preparation

We collected serum and bone specimens from 40 elderly patients with fracture and 21 age-matched patients without fracture at the age between 60 and 90 years old from three hospitals (China-Japan Friendship Hospital in Beijing, Shanghai Guanghua Rheumatic Hospital and Shenzhen People's Hospital; Supplementary Table 2). Either elderly postmenopausal women with low-energy fractures (that is, osteoporotic fractures) or age-matched women undergone revision total hip/knee arthroplasty without fracture history were included (inclusive criteria). Patients with diabetes, malignancy, coronary heart disease or other severe diseases in the previous 5 years were excluded in our study (exclusive criteria). The Committees of Clinical Ethics of the three hospitals approved the sample collection procedures, which conformed to the principles of the Helsinki Declaration. We also obtained informed consent from the participants.

### Preparation of osteoclasts

We prepared and cultured mouse osteoclasts according to the previously reported protocols^35^. Briefly, the bone marrow cells were isolated from the long bones (tibia and femur) of 8- to 12-week-old female mice and cultured in α–MEM containing 10% FBS and 1% penicillin–streptomycin for 24 h to generate BMMs. To produce osteoclasts for the subsequent experiments, BMMs were cultured on tissue culture plastic or coverslips in α–MEM for 2 days with M–CSF (25 ng ml^−1^; PeproTech), and for an additional 6 days in the same medium with 25 ng ml^−1^ recombinant mouse M–CSF and 5 ng ml^−1^ recombinant mouse RANKL (PeproTech) to avoid contamination with osteoblasts. The culture medium was replaced every 2 days. To prepare and culture human osteoclasts, we obtained peripheral blood samples from healthy donor under a protocol approved by the Committees of Clinical Ethics of the above hospitals. Then, the PBMCs were isolated by Ficoll-Paque (Amersham Biosciences) density gradient centrifugation and plated at 10^6^ cells per cm^2^, and nonadherent cells were removed by washing in PBS. The cells were cultured in α–MEM containing 10% FBS and 1% penicillin–streptomycin. Then, the PBMCs were cultured in the above medium supplemented with 25 ng ml^−1^ recombinant human M-CSF (PeproTech) and 30 ng ml^−1^ recombinant human RANKL (PeproTech) for 7 days to induce osteoclast formation. The purified osteoclasts (OSCAR^+^ cells) were isolated by MACS using anti-OSCAR antibody (R&D, Rat IgG, MAB1633) in combination with anti-Rat IgG MicroBeads (Miltenyi Biotec).

### Preparation of osteoblasts

Primary osteoblast precursor cells were isolated from the calvarial bone of newborn C57BL/6 mice (1- to 2-day-old) through enzymatic digestion with α-MEM containing 0.1% collagenase (Life technologies) and 0.2% dispase II (Life Technologies). The isolated osteoblast precursor cells were promoted with osteogenic α-MEM medium with 10% FBS, 1% penicillin–streptomycin, 5 mM β-glycerol phosphate (Sigma), 0.1 mg ml^−1^ ascorbic acid (Sigma) and 10 nM dexamethasone (Sigma) for 9 days and culture medium was replaced every 2–3 days^36^. The human primary osteoblasts (human OBs) were purchased from PromoCell. The human OB cells were cultured and promoted with osteoblast growth medium and osteoblast mineralization medium (PromoCell), respectively. The purified osteoblasts (ALP^+^ cells) were isolated by MACS using anti-ALP antibody (Abcam, Rabbit IgG, Ab108337) in combination with anti-Rabbit IgG MicroBeads (Miltenyi Biotec).

### Co-culture and transfection experiments

The well inserts with a 0.4-μm pore-sized filter (Greiner) for six-well plates were used following the manufacturer's instructions. Primary osteoblast precursor cells were seeded into the well inserts and differentiated into osteoblasts with osteogenic α-MEM medium. BMMs were seeded into six-well plates and induced into osteoclasts by complete α–MEM containing M–CSF and RANKL. After differentiation, osteoblasts and osteoclasts were washed with PBS, and then co-cultured for different time according to the experimental requirements. All co-culture experiments were done in complete α-MEM with exosome-free FBS (Life Technologies). The lentiviral vector expressing ATF4 3′UTR (ATF4 3′UTR)/Negative control lentivirus (3′UTR NC), lentiviral vector encoding red fluorescence protein (LV-RFP) and CMV-GFP-CD63 lentiviral vector (GFP-CD63) were purchased from Shanghai Integrated Biotech Solutions Co., Ltd. In the case of ATF4 3′UTR (3′UTR NC)/GFP-CD63-transfected osteoclasts and LV-REP-transfected osteoblasts, the cells were transfected at a multiplicity of infection (MOI) of 100 the day before the co-culture experiment according to the manufacturer's instructions.

### *miR-214-3p*-depleted MC-3T3-E1 cells and RAW264.7 cells

A CRISPR/Cas9 system targeting *mmu-miR-214-3p* gene was purchased from Shanghai Integrated Biotech Solutions Co., Ltd. The 20-nt guide sequences targeting mouse mmu-*miR-214-3p* was designed and cloned into a Cas9-2A-Puro plasmid containing a mouse CMV promoter-driven Cas9 expression cassette. MC-3T3-E1 cells/RAW264.7 cells cultured in DMEM medium were transfected with Cas9-2A-Puro control and mmu-*miR-214-3p* gene-targeted gRNA-containing plasmids using Lipofectamine 2000 (Life technologies). After 24 h incubation, cells were changed to fresh complete medium containing 1 μg ml^−1^ puromycin for 48 h. Then, the cells were changed to fresh complete DMEM medium for 24 h and subsequently split to clonal density. After ∼10 days, clonal cells were picked and expanded for analysis. Cloning of PCR products purified from individual clones was performed using pGEM-T Easy Vector Systems (Promega, Cat no. A1380). Mutants were identified by Sanger sequencing (Sangon). The mmu-*miR-214-3p* gRNA oligonucleotides were as follows: forward, 5′-ACCGTGCCTGCTGTACAGGTGAG-3′, reverse, 5′-AAACCTCACCTGTACAGCAGGCA-3′.

### Genetic mouse model

To generate the OC-miR-214-3p mice, first, we generate the *Rosa26-PCAG-STOP*^*fl*^-*mmu-miR-214-3p*-knock-in mice. In brief, a cassette containing the following components was constructed to target the *Rosa26* locus: *FRT-LoxP-stop* codons-three SV40 *poly(A)* sequences-*LoxP-mmu-miR-214-3p-WPRE-bGH poly(A)-AttB-PGK* promoter-*FRT-Neo-PGK poly(A)-AttP* (Fig. 2a). The targeting vector is constructed, fully sequenced and electroporated into C57BL/6 embryonic stem cells. Positive targeting clones were identified by PCR and southern blotting. The targeted embryonic stem clones were microinjected into BALB/c blastocysts to obtain chimeric mice following standard procedures. Thereafter, the chimeric mice were intercrossed with C57BL/6 mice to obtain F1 heterozygote mice and then backcrossed with C57BL/6 mice to expand the enough number of heterozygote *Rosa26-PCAG-STOP*^*fl*^-*mmu-miR-214*-knock-in mice. Thereafter, we crossed the *Rosa26-PCAG-STOP*^*fl*^-*mmu-miR-214*-knock-in mice with *Ctsk-Cre* mice to obtain OC-miR-214-3p mice. The littermates were used as WT control. The *Ctsk*-Cre mouse was a generous gift from Professor Xu Jiake in the West Australia University. It is the offspring of the *Ctsk*-Cre mouse strain established by the Shigeaki Kato's lab^42^. The genetically modified mice were all maintained under standard animal housing conditions (12-h light, 12-h dark cycles and free access to food and water). We obtained animal ethics approval from the Committees of Animal Ethics and Experimental Safety of Hong Kong Baptist University.

### OVX-induced osteoporotic mouse model

The female C57BL/6 mice were maintained under standard animal housing conditions (12-h light, 12-h dark cycles and free access to food and water). The female mice were OVX or sham-operated at 6 months of age. At the corresponding time points in each study, the OVX mice were euthanized for collecting serum and bilateral femurs and tibias for the subsequent *ex vivo* analysis. All the experimental procedures were approved by the Committees of Animal Ethics and Experimental Safety of Hong Kong Baptist University.

### Osteoclast-targeting delivery system containing antagomiR-214-3p

AntagomiR-214-3p was encapsulated into D-Asp_8_-liposome using our previously established protocol^33^. Briefly, the lipids of DOTAP, DOPE, Chol, DSPE-mPEG2000 and DSPE-PEG2000-MAL at a molar ratio of 42:15:38:3:2 dissolved in chloroform were dried into a thin film and hydrated with PBS. The dispersion was then extruded in a LipoFast mini extruder through two stacked polycarbonate membranes (0.2 and 0.1 μm) in stepwise manner. Then, the preformed liposome was incubated with D-Asp_8_ peptide with an N-terminal acetylcysteine residue for 2 h at ambient temperature. Subsequently, the liposome suspension was purified by Sepharose CL-4B column to remove the un-conjugated D-Asp_8_ peptide. The liposome suspension in 0.5 ml aliquots were mixed with 0.5 ml distilled water containing mannitol and lyophilized for 48 h. Finally, the above lyophilized liposomes with 15 μmol lipids were rehydrated by adding 0.5 ml DEPC-treated water containing antagomiR-214-3p (750 μg) and incubated for 20 min at room temperature. The encapsulation procedure was performed immediately before use and then sterilized by passing through a 0.22-μm sterile filter.

### Serum exosomes and supernatant exosomes isolation

A multi-step centrifugation procedure was used to isolate exosomes as described previously^43^. The collected serum and culture supernatant were pre-purified by centrifugation at 300*g* for 10 min at 4 °C to remove floating cells, followed by a second centrifugation at 820*g* for 15 min, 10,000*g* for 5 min at 4 °C and passage through a 0.8-μm syringe filter to remove cell debris. The extracellular exosomes (size, <1 μm) were pelleted in a final centrifugation at 100,000*g* for 2 h at 4 °C using an SW28 rotor (Beckman). Pelleted exosomes were resuspended in PBS and ultracentrifuged again. The protein concentration in exosomes was determined by BCA protein assay kit (Thermo Scientific, Product # 23225). The size distribution of exosomes was examined using a NanoSight Tracking Analysis LM20 System (NanoSight Ltd.).

### Nanoparticle tracking analysis

Nanoparticle tracking analysis measurements were performed with a NanoSight LM20 (NanoSight), equipped with a sample chamber with a 640-nm laser and a Viton fluoroelastomer O-ring. The samples were diluted at ∼2–5 ng μl^−1^ and injected in the sample chamber with sterile syringes (BD Discardit II) until the liquid reached the tip of the nozzle. All measurements were performed at room temperature.

### FACS of osteoblasts

Bone marrow cells were harvested from bilateral femur of the mice. Goat polyclonal alkaline phosphatase (ALP) primary antibody (1:50, R&D systems, AF2910) and phycoerythrin (PE)-conjugated anti-goat IgG secondary antibody (1:200, R&D systems, F0107) were used to stain the ALP^*+*^ populations. Briefly, after washing with PBS and incubating with 1% BSA, the bone marrow cells were stained with the Anti-Mouse ALP polyclonal antibody. Then, the stained cell populations were washed three times and subsequently incubated with PE-conjugated secondary antibody. Finally, the incubated cells were washed, then sorted and analysed by FACS Aria II Flow Cytometer (BD Biosciences). The selected ALP^*+*^ cells were used for total RNA extraction followed by real-time PCR analysis^44^,^45^.

### Magnetic-activated cell sorting

Bone marrow cells were flushed from the diaphysis of the femur and dissociated to a single-cell suspension by pipetting up and down and passed through 30 μm nylon mesh to remove cell clumps, which may clog the column. Cultured osteoclasts were trypsinized and collected. Cells were centrifuged at 300*g* for 10 min and 10^7^ total cells were re-suspended in 200 μl of ice-cold buffer (Dulbecco's phosphate buffered saline without Ca^2+^ and Mg^2+^, with 0.5% bovine serum albumin and 2 mM EDTA). 10 μg ml^−1^ Rat monoclonal anti-OSCAR (R&D Systems MAB1633) was added to the cell suspension and incubated at 4 °C for 1 h. After washing and centrifuge, the cells were re-suspended in 80 μl of ice-cold buffer. 20 μl of Anti-Rat IgG MicroBeads (Miltenyi Biotec, #130-048-501) was added per 10^7^ total cells, incubated for 15 min at 4 °C. Cells were washed with 1–2 ml of buffer per 10^7^ cells, centrifuged at 300*g* for 5 min, and re-suspended in 500 μl of buffer. A MACS column (Miltenyi Biotec) was placed in the magnetic field of a suitable MACS Separator (Miltenyi Biotec) and prepared by rinsing with 3 ml of buffer. The column was washed by 3 ml of buffer for three times. Flow-through of unlabelled cells (OSCAR^−^ cells) was collected. The column was placed over a collection tube, 5 ml buffer was pipetted onto the column, and the magnetically labelled cells (OSCAR^+^ cells) were flushed out by firmly pushing the plunger into the column. OSCAR^+^ and OSCAR^−^ cells were collected for downstream analysis. To isolated the ALP^+^ and ALP^−^ cells in cultured osteoblasts, 10 μg ml^−1^ Rabbit monoclonal anti-ALP (Abcam ab108337) and 20 μl Anti-Rabbit IgG MicroBeads (Miltenyi Biotec, #130-048-602) were was added per 10^7^ total cells and sorted as above mentioned.

### Laser captured micro-dissection

The femur was decalcified in 10% EDTA and embedded in optimal cutting temperature compound (OCT). Then, the series frozen sections (5 μm) from distal femur were prepared in a cryostat (Leica Microsystems) at −24 °C. The adjacent sections were mounted on either polyethylene membrane-equipped slides (P.A.L.M.) or glass slides. The sections mounted on glass slides were performed immunostaining to identify the CTSK^*+*^ and ALP^+^ cells, respectively. Briefly, the cryosections were incubated overnight at 4 °C with rabbit polyclonal anti-CTSK antibody (1:50, Abcam, ab19027) or goat polyclonal anti-ALP antibody (1:50, R&D systems, AF2910) after fixation and blocking. Then, the sections were incubated with Alexa Fluor 488 donkey anti-rabbit IgG (H+L) Antibody (1:400, Thermo Fisher Scientific, A-21206) or PE-conjugated anti-goat IgG (1:400, R&D systems, F0107). Finally, the sections were mounted with medium containing DAPI (Sigma) and examined under a fluorescence microscope to identify the CTSK^+^/ALP^+^ staining cells. The adjacent sections mounted on membrane-coated slides were stained with neutral red for 1 min at room temperature. After brief rinsing in water, the sections were air-dried. CTSK^*+*^/ALP^+^ staining cells in adjacent sections were isolated by micro-dissection with an upgraded laser pressure catapulting micro-dissection system (P.A.L.M.) using a pulsed 355 nm diode laser (LMD 7000, Leica). About 150 identified cells were collected in reaction tube containing 5 μl QIAzol Lysis Reagent (miRNeasy Micro Kit, Qiagen) for total RNA extraction and subsequent real-time PCR analysis^23^.

### RT–PCR and real-time PCR

Total RNA in the cells or bones and total miRNAs in serum or exosomes were isolated using TRIzol (Life technologies) reagent and miRNeasy Serum/Plasma Kit (Qiagen) or mirVana miRNA Isolation Kit (Ambion) according to the manufacturer's protocol, respectively. After purification, the total RNA was treated with TURBO DNase (Ambion) and reverse transcribed into first-strand cDNA using a high-capacity cDNA reverse transcription kit (Applied Biosystems). We used 100 ng cDNA per PCR. The TaqMan primer-probe combinations for pri-miRNAs, pre-miRNAs and miRNAs were products of Ambion. The primer for mRNAs were purchased from Shanghai Integrated Biotech Solutions Co., Ltd (Supplementary Table 6). For detection of 3′ end uridylated miR-214-3p isoforms, the following miRNA assays were used: hsa-miR-214-3p (Ambion, ID 002306), hsa-miR-214-3p-AAA (custom order: 5′-ACAGCAGGCACAGACAGGCAGUAAA-3′, hsa-miR-214-3p-UUU (custom order: 5′-ACAGCAGGCACAGACAGGCAGUUUU-3′). Real-time PCR reactions were performed using the 96-well ABI Prism 7900 HT Sequence detection system (Applied Biosystems). Please see Supplementary Table 5 for the raw data for all the real-time PCR analysis.

### Western blot analysis

Proteins were extracted from the ultracentrifugation pellets and separated on a denatured SDS–polyacrylamide gel before transfer to a polyvinylidene difluoride membrane. The blotting membrane was blocked with bovine serum albumin and incubated with goat polyclonal anti-Sema4D antibody (1:100; Santa Cruz, sc-16632), rabbit polyclonal anti-CTSK antibody (1:100; Santa Cruz, sc-30056), goat polyclonal anti-tartrate-resistant acid phosphatase antibody (1:100; Santa Cruz, sc-30833), rabbit polyclonal anti-CD9 antibody (1:100; Santa Cruz, sc-9148), rabbit polyclonal anti-CD63 antibody (1:100; Santa Cruz, sc-15363), rabbit polyclonal anti-Flotillin-1 antibody (1:100; Santa Cruz, sc-25506), goat polyclonal anti-TSG101 antibody (1:100; Santa Cruz, sc-6037) and rabbit polyclonal anti-calnexin antibody (1:100; Santa Cruz, sc-11397) followed by incubation with horseradish peroxidase (HRP)-coupled goat anti-rabbit IgG H&L (1:5,000, Abcam, ab6721) or HRP-coupled rabbit anti-goat IgG H&L (1:5,000, Abcam, ab6741), respectively. The proteins were detected using SuperSignal West Dura Extended Duration Substrate (Thermo Scientific, Prod # 34075). Supplementary Fig. 17 depicts uncropped western blots.

### Biophotonic imaging analysis on organ distribution of exosomes

Fluorescence imaging for PKH67-labelled exosomes in these organs was performed using an IVIS 200 imaging system (Caliper Life Sciences). Briefly, the mice were feed with alfalfa-free diet for 3–5 days before the study. After exosomes isolation from the culture medium of OC-miR-214-3p osteoclasts differentiated from OC-miR-214-3p mice-derived BMMs, the isolated exosomes were pre-labelled using PKH67 Green Fluorescent Cell Linker Kit (Sigma). Then, the PKH67 exosomes (100 μg per mouse) were intravenously injected into 12-week-old female C57BL/6 mice. Thereafter, the mice were killed at 4 and 8 h after the injection and subjected to biophotonic imaging, respectively. Identical illumination settings, including exposure time (10 s), binning factor (4), f-stop (2) and fields of view (10 cm for width and length, respectively), were used for all image acquisition.

### Immunohistochemistry

The femur samples were fixed with 4% paraformaldehyde and embedded with OCT after decalcification with 10% EDTA. The frozen frontal sections (5 μm) were cut in a freezing cryostat at −20 °C. The sections were air dried at room temperature, fixed in ice-cold acetone for 10 min, permeablilized with 0.1% Triton X-100 at room temperature for 20 min and blocked in 5% donkey serum in PBS. The sections were then incubated overnight at 4 °C with either goat polyclonal anti-ALP antibody (1:50, R&D systems, AF2910) or rabbit polyclonal anti-CTSK antibody to Cathepsin K (1:50, Abcam, ab19027). Following three washes in PBS, the sections were incubated with PE-conjugated anti-goat IgG (1:400, R&D systems, F0107) or Alexa Fluor 555-conjugated anti-rabbit IgG (1:400, Thermo Fisher Scientific, A-21428) secondary antibody (R&D systems, F0107) for 1 h. Negative control sections were set by omitting the primary antibodies. The sections were mounted with the medium containing DAPI (Sigma) and examined under either fluorescence microscope (80i, Nikon) or confocal laser scanning microscope (TCS SP8, Leica)^46^.

### Micro-CT

The femurs isolated from the mice were scanned by micro-CT system (viva CT40, SCANCO MEDICAL) according to our established protocols^23^. Briefly, 426 slices with a voxel size of 10 μm were scanned at the region of the distal femur beginning at the growth plate and extending proximally along the femur diaphysis. Eighty continuous slices beginning at 0.1 mm from the most proximal aspect of the growth plate in which both condyles were no longer visible were selected for analysis. The whole trabecular bone were isolated for three-dimensional reconstruction (Sigma=1.2, Supports=2 and Threshold=180) to calculate the following parameters: BMD, relative bone volume (BV/TV), trabecular number (Tb.N) and trabecular thickness (Tb.Th).

### Bone histomorphometry

The distal femur was dehydrated in graded concentrations of ethanol and embedded without decalcification in the modified methyl methacrylate using our previously established protocol^47^. After dehydration, frontal sections for trabecular bone were obtained from the distal femur at a thickness of 15 mm with Leica SM2500E microtome (Leica Microsystems). Modified Masson's trichrome and Tartrate-resistant acid phosphatase staining of the sections were performed for analysis of static parameters, or the sections were left unstained for collection of fluorochrome-based data. Bone dynamic histomorphometric analyses for MAR, BFR/BS and bone static histomorphometric analyses for osteoblast surface (the percent of trabecular bone surface covered by osteoblasts, Ob.S/BS), osteoblast number per bone perimeter (Ob.N/B.Pm), osteoclast surface (the percent of trabecular bone surface covered by osteoclasts, Oc.S/BS) and osteoclast number per bone perimeter (Oc.N/B.Pm) were made using professional image analysis software (Image J, NIH) under fluorescence microscope (Leica image analysis system, Q500MC). The bone histomorphometric parameters were calculated and expressed according to the standardized nomenclature for bone histomorphometry.

### Statistical analysis

In general, statistical differences among groups were analysed by one-way analysis of variance (ANOVA) with a *post-hoc* test (after normalization to baseline/control group) to determine group differences in the study parameters. Especially, the rescue treatment data were compared by means of two-way ANOVA (genetic background and pharmacological treatment) with full factorial design. When ANOVA revealed differences and when treatment presented homogeneity of variance, we performed Tukey's honestly significant difference test for multiple comparison. For heterogeneous variance, we employed Games-Howell multiple comparison tests. All statistical analyses were performed with SPSS software at version 18.0 (SPSS Inc.).

### Power analysis

The calculation of the sample size in this study was based on the following formula:


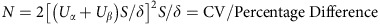


For study 2, according to our pilot data in a small scale, it showed a percentage difference of 10.4% at least in ‘bone formation rate' between the OC-miR-214-3p mice and WT controls. In addition, 5% of coefficient of variation (CV) for measurement repeatability of ‘BFR/BS' was found. So, a sample size of *n*=6 in each group will be enough to give 1-*β*=90% power (*β*=10%, *u*_β_=1.28) to detect a 10.4% difference, assuming a CV of 5% for ‘BFR/BS' and setting *α* at 0.05 (*u*_α_=1.96). For studies 4 and 5, based on our pilot data with a small sample size, it showed a percentage difference of 7.6% at least in ‘BFR/BS' between OC-miR-214-3p exosomes-treated mice and WT exosomes-treated mice or between the OVX mice in AMO and NC group. In addition, 5% of CV for measurement repeatability of ‘BFR/BS' was found. So, a sample of *n*=9 in each group will be enough to give 1-*β*=90% power (*β*=10%, *u*_β_=1.28) to detect a 7.6% difference, assuming a CV of 5% for ‘bone formation rate' and setting *α* at 0.05 (*u*_α_=1.96).

## Additional information

**How to cite this article:** Li, D. *et al.* Osteoclast-derived exosomal miR-214-3p inhibits osteoblastic bone formation. *Nat. Commun.* 7:10872 doi: 10.1038/ncomms10872 (2016).

## Supplementary Material

Supplementary Information Supplementary Figures 1-17 and Supplementary Tables 1-6.
