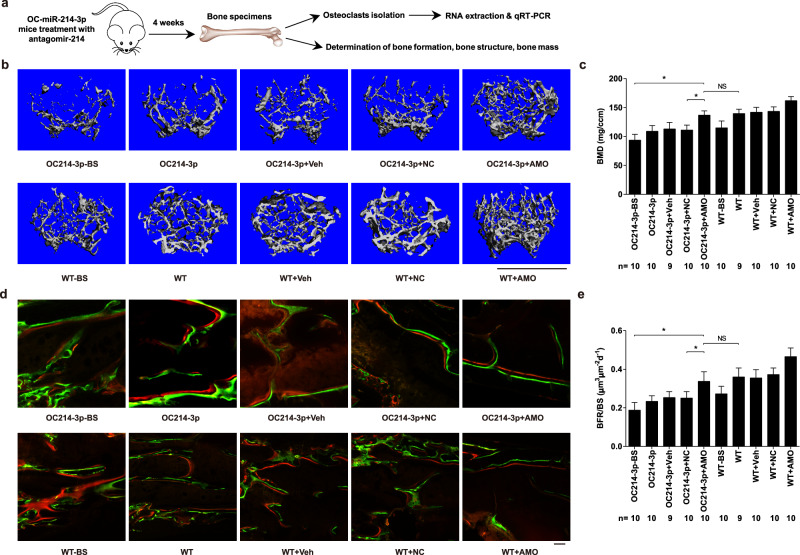# Author Correction: Osteoclast-derived exosomal miR-214-3p inhibits osteoblastic bone formation

**DOI:** 10.1038/s41467-025-61082-9

**Published:** 2025-06-19

**Authors:** Defang Li, Jin Liu, Baosheng Guo, Chao Liang, Lei Dang, Cheng Lu, Xiaojuan He, Hilda Yeuk-Siu Cheung, Liang Xu, Changwei Lu, Bing He, Biao Liu, Atik Badshah Shaikh, Fangfei Li, Luyao Wang, Zhijun Yang, Doris Wai-Ting Au, Songlin Peng, Zongkang Zhang, Bao-Ting Zhang, Xiaohua Pan, Airong Qian, Peng Shang, Lianbo Xiao, Baohong Jiang, Chris Kong-Chu Wong, Jiake Xu, Zhaoxiang Bian, Zicai Liang, De-an Guo, Hailong Zhu, Weihong Tan, Aiping Lu, Ge Zhang

**Affiliations:** 1 Institute for Advancing Translational Medicine in Bone and Joint Diseases, School of Chinese Medicine, Hong Kong Baptist University, Hong Kong SAR, 999077 China; 2 Institute of Integrated Bioinfomedicine and Translational Science, School of Chinese Medicine, Hong Kong Baptist University, Hong Kong SAR, 999077 China; 3 Shenzhen Lab of Combinatorial Compounds and Targeted Drug Delivery, HKBU Institute of Research and Continuing Education, Shenzhen, 518057 China; 4 Research Group of Bone and Joint Diseases, HKBU Institute of Science and Technology, Haimen, 226100 China; 5 Academician Chen Xinzi Workroom for Advancing Translational Medicine in Bone and Joint Diseases, Kunshan RNAi Institute, Kunshan Industrial Technology Research Institute, Kunshan, Jiangsu 215300 China; 6 Shum Yiu Foon Shum Bik Chuen Memorial Centre for Cancer and Inflammation Research, Hong Kong Baptist University, Hong Kong SAR, 999077 China; 7 Hong Kong Baptist University Branch of State Key Laboratory of Chemo/Biosensing and Chemometrics of Hunan University, Hong Kong, 999077 China; 8 Hong Kong Baptist University–Northwestern Polytechnical University Joint Research Centre for Translational Medicine on Musculoskeletal Health in Space, Shenzhen, 518057 China; 9 Institute of Basic Research in Clinical Medicine, China Academy of Chinese Medical Sciences, Beijing, 100700 China; 10 Department of Biology and Chemistry, City University of Hong Kong, Hong Kong SAR, 999077 China; 11 Department of Spine Surgery, Shenzhen People’s Hospital, Ji Nan University Second College of Medicine, Shenzhen, 518020 China; 12 School of Chinese Medicine, Faculty of Medicine, Chinese University of Hong Kong, Hong Kong SAR, 999077 China; 13 Department of Orthopaedics and Traumatology, Bao’an Hospital Affiliated to Southern Medical University and Shenzhen 8th People Hospital, Shenzhen, 518100 China; 14 Key Laboratory for Space Bioscience and Biotechnology, Institute of Special Environmental Biophysics, School of Life Science, Northwestern Polytechnical University, Xi’an, 710072 China; 15 Institute of Arthritis Research, Shanghai Academy of Chinese Medical Sciences, Shanghai, 200052 China; 16 Shanghai Institute of Materia Medica, Chinese Academy of Sciences, Shanghai, 201203 China; 17 Department of Biology, Hong Kong Baptist University, Hong Kong SAR, 999077 China; 18 Molecular Laboratory, School of Pathology and Laboratory Medicine, University of Western Australia, Nedlands, Western Australia 6907 Australia

Correction to: *Nature Communications* 10.1038/ncomms10872, published online 7 March 2016

In the version of the article initially published, the microscopic image of ‘WT’ group in Fig. 3h was identical to the image of ‘WT+Veh’ group in Fig. 4d. The microCT image of ‘OC214-3p+Veh’ group in Fig. 4b was chosen from a wrong sample. We confirm that the inadvertent image duplication and misplacement occurred due to improper naming when exporting the original imaging data, which have now been replaced by the correct images in the corresponding figures (see Figs. 1 and 2 below). Raw data for both Figs. 3 and 4 can be accessed at 10.6084/m9.figshare.28915727.v1. The error and corrections do not alter the significance of findings or conclusions of this study. We apologize for any confusion these mistakes may have created.

Fig. 1 Corrected Fig. 3
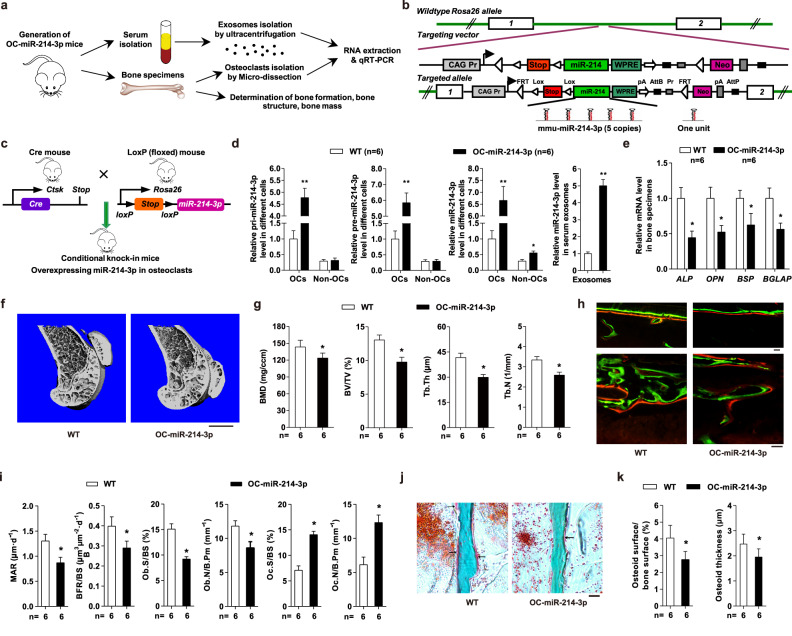


Fig. 2 Corrected Fig. 4