# Supplementary material for: Osteoclast-derived exosomal miR-214-3p inhibits osteoblastic bone formation
Source: Nat Commun. 2016 Mar 7;7:10872. doi: 10.1038/ncomms10872 (PMC4786676; doi:10.1038/ncomms10872)
Supplement: Supplementary Information — Supplementary Figures 1-17 and Supplementary Tables 1-6. [file ncomms10872-s1.pdf]

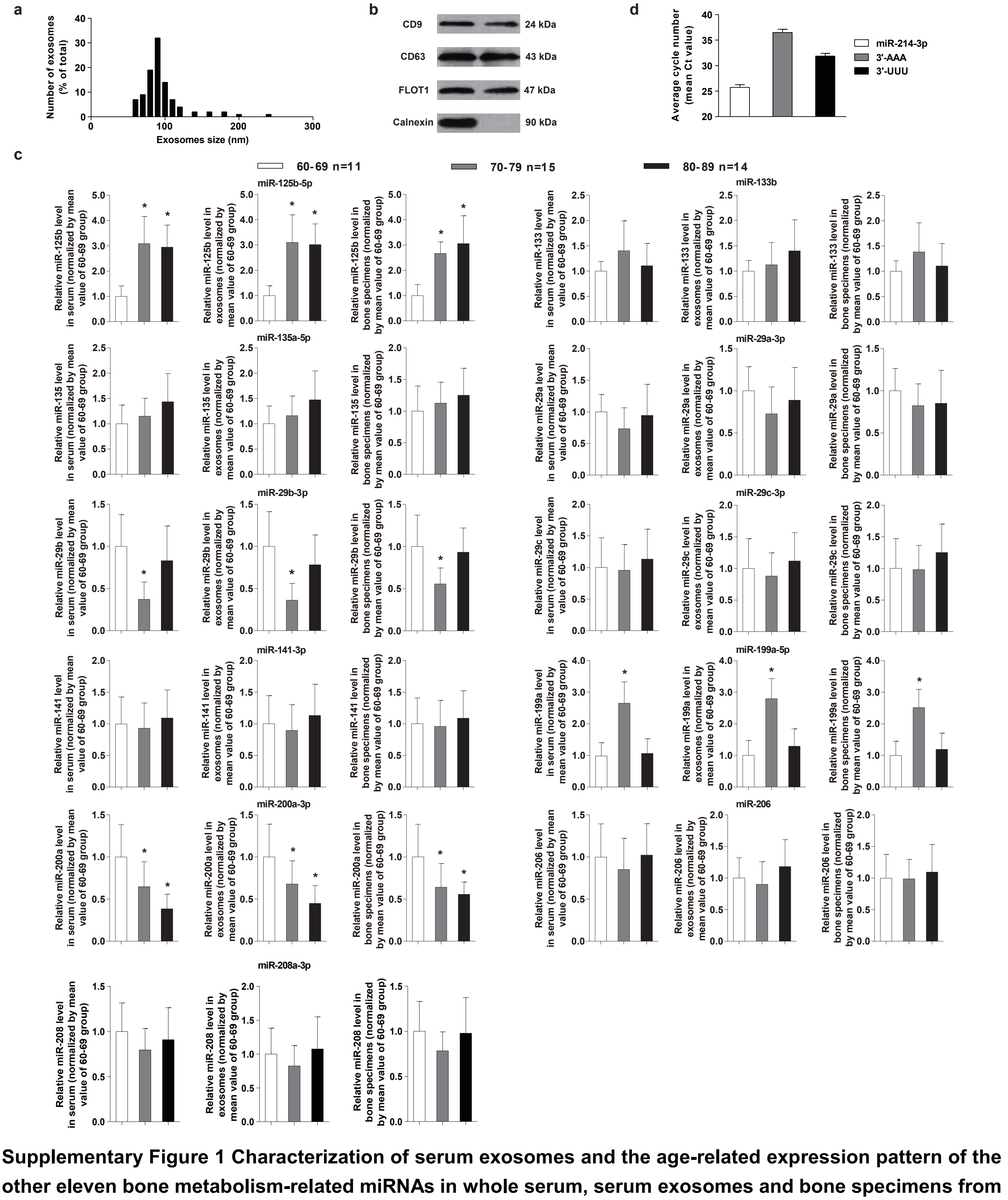

Supplementary Figure 1 Characterization of serum exosomes and the age-related expression pattern of the other eleven bone metabolism-related miRNAs in whole serum, serum exosomes and bone specimens from elderly patients with fractures. (**a**) The size distribution of serum exosomes. (**b**) Western blot analysis of the exosomal membrane markers (CD9, CD63 and FLOT1) and Calnaxin (a cytoplasmic protein not present in exosomes in the lysates of ultracentrifugation pellets isolated from whole serum. (**c**) Real-time PCR analysis of the age-related changes in the other eleven miRNAs levels in whole serum (left panel), serum exosomes (middle panel) and bone specimens (right panel) from elderly patients with fractures, respectively. (**d**) Real-time PCR analysis of the expression of canonical miR-214-3p, 3' end adenylated miR-214-3p (3'-AAA), and 3' end uridylated miR-214-3p (3'-UUU) in exosomal RNA fractions by TaqMan microRNA assays and Custom TaqMan small RNA assays. Note: All data were presented as the mean ± s.d. The relative miRNAs or mRNA levels were normalized to the mean value of the 60-69 group. Human RUN6B are used as the internal control. Data are the mean ± s.d. **P* < 0.05 *v.s.* 60-69 group. One-way ANOVA with a *post-hoc* test was performed.

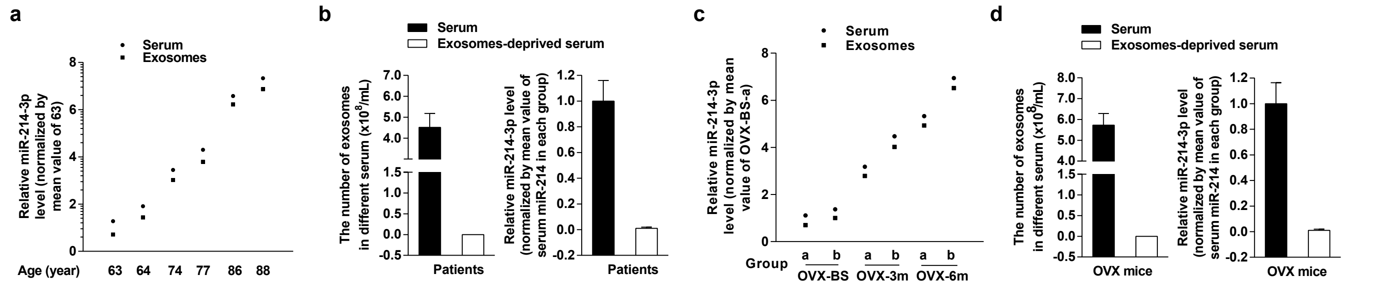

Supplementary Figure 2 The serum miR-214-3p is concentrated in serum exosomes. (**a**) Real-time PCR analysis of the relative miR-214-3p levels in serum and serum exosomes from elderly patients with fractures. (**b**) The number of exosomes and the miR-214-3p levels in serum and exosomes-deprived serum from elderly patients with fractures. **(c)** Real-time PCR analysis of the relative miR-214-3p levels in serum and serum exosomes from OVX mice. **(d)** The number of exosomes and the miR-214-3p levels in serum and exosomes-deprived serum from the OVX mice. Equal volume of serum was used in each test. All data are the mean ± s.d.

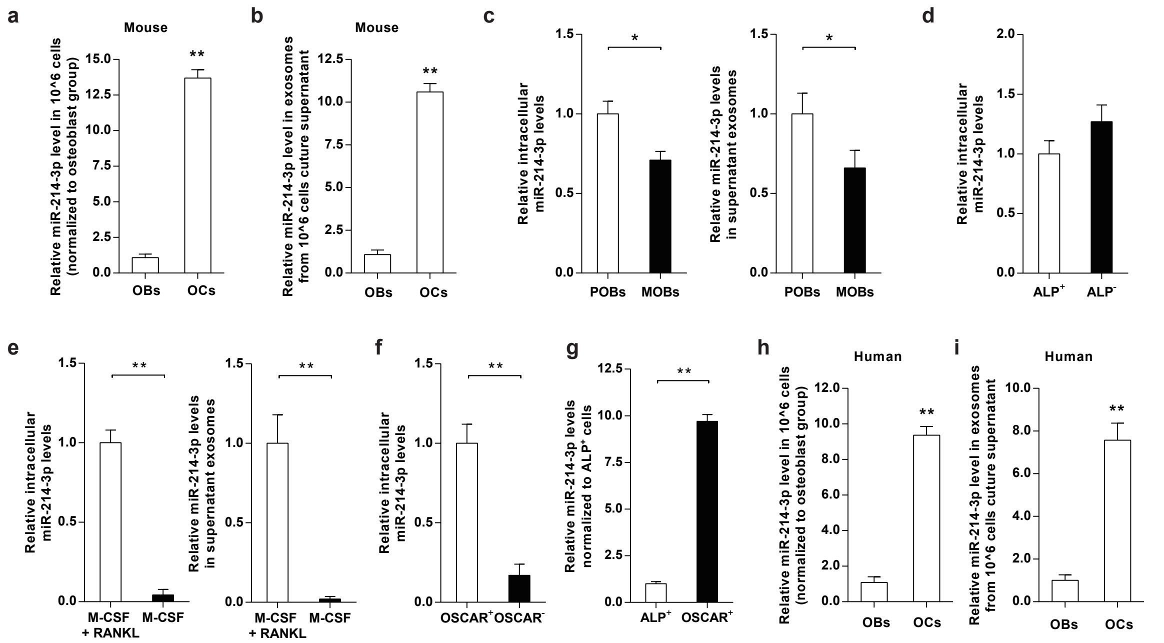

Supplementary Figure 3 miR-214-3p is abundant in osteoclasts / the osteoclast-derived supernatant exosomes rather than in osteoblasts / the osteoblast-derived supernatant exosomes. (**a**) Real-time PCR analysis of the miR-214-3p levels in mouse osteoblasts (mouse OBs) differentiated from calvarial bone-derived osteoblast precursor cells (POBs) and osteoclasts (mouse OCs) differentiated from bone marrow macrophages (BMMs). (**b**) Real-time PCR of the levels of exosomal miR-214-3p in the supernatant of mouse OBs and mouse OCs, respectively. (**c**) Real-time PCR analysis of the miR-214-3p levels in cells and supernatant exosomes from the POBs or mature osteoblasts (MOBs) differentiated from POBs in osteogenic medium for 9 days. (**d**) Real-time PCR analysis of the miR-214-3p levels in ALP^+ (purified osteoblasts) and ALP^- cells isolated from the calvarial bone-derived osteoblast precursor cells after stimulation with osteogenic medium for 9 days by magnetic-activated cell sorting (MACS). The ALP^+ cells were isolated by anti-ALP antibody (Rabbit IgG) in combination with anti-Rabbit IgG MicroBeads. (**e**) Real-time PCR analysis of the miR-214-3p levels in cells and supernatant exosomes from the BMMs at day 7 after induction either by M-CSF (30 ng/ml) alone or by M-CSF (30 ng/ml) and RANKL (5 ng/ml). (**f**) Real-time PCR analysis of the miR-214-3p levels in $OSCAR^+$ (purified osteoclasts) and $OSCAR^-$ cells isolated from the BMMs at day 7 after induction by M-CSF (30 ng/ml) and RANKL (5 ng/ml) by MACS. The $OSCAR^+$ cells were isolated by anti-OSCAR antibody (Rat IgG) in combination with anti-Rat IgG MicroBeads. (**g**) Real-time PCR analysis of the miR-214-3p levels in either $OSCAR^+$ cells (purified osteoclasts) or ALP^+ cells (purified osteoblasts). (**h**) Real-time PCR analysis of the levels of miR-214-3p in human primary osteoblasts (human OBs) and human osteoclasts (human OCs) differentiated from human peripheral blood mononuclear cells. (**i**) Real-time PCR analysis of the levels of exosomal miR-214-3p in the supernatant of human OBs and human OCs. All data are the mean ± s.d. of 4 independent experiments. *P<0.05. **P<0.01. Student's t test was performed.

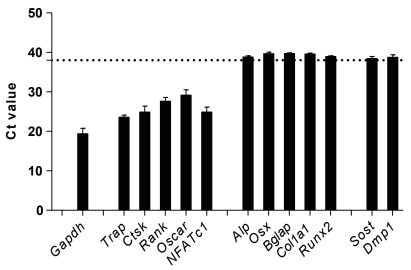

Supplementary Figure 4 The expression of osteoclast/osteoblast/osteocyte marker genes in CTSK$^+$ cells isolated from femur cryosections by laser-captured microdissection. Real-time PCR analysis of the *Ct* value of osteoclast marker genes (*Trap*, *Ctsk*, *Rank*, *Oscar* & *NFATc1*), osteoblast marker genes (*Alp*, *Osx*, *Bglap*, *Col1a1* & *Runx2*), osteocyte marker genes (*Sost* & *Dmp1*) and reference gene (*Gapdh*). Dotted line indicates the *Ct* value of 38. Data are the mean ± s.d.

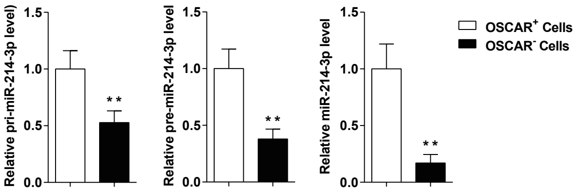

Supplementary figure 5 Pri-miR-214-3p, pre-miR-214-3p and mature miR-214 are abundant in osteoclasts rather than the other bone marrow cells. Real-time PCR analysis of the levels of pri-miR-214-3p, pre-miR-214-3p and mature miR-214 in either Osteoclasts ($OSCAR^+$) or non-osteoclasts ($OSCAR^-$ cells), respectively. $OSCAR^+$/$OSCAR^-$ cells were isolated from whole bone marrow of aging OVX mice by magnetic-activated cell sorting using anti-OSCAR antibody (Rat IgG) in combination with anti-Rat IgG MicroBeads. All data are the mean ± s.d. of 6 biological replicates. $^{**}P<0.01$. Student's *t* test was performed.

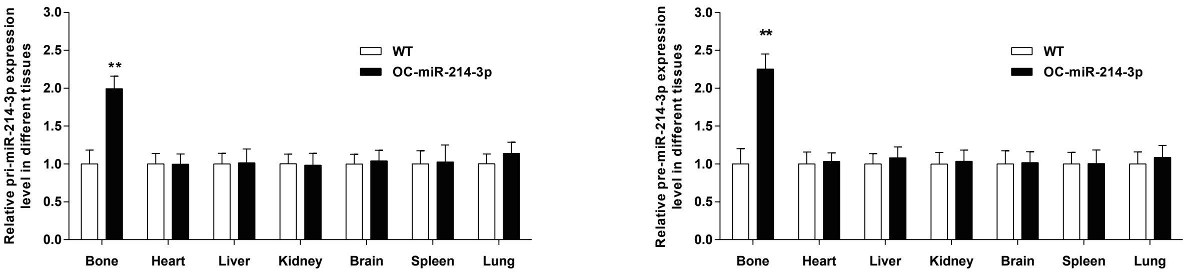

Supplementary Figure 6 Characterization of pri-miR-214-3p and pre-miR-214-3p expression in OC-miR-214-3p mice. Real-Time PCR of the pri-miR-214-3p and pre-miR-214-3p levels in bone and other tissues (normalized to the mean values of WT mice) from OC-miR-214 and WT mice, respectively. *n*=6 for each group. All data are the mean ± s.d. ***P* < 0.01 *vs.* WT. Student's *t* test was performed.

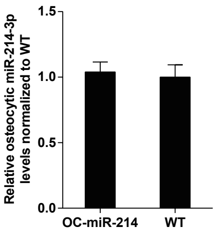

Supplementary figure 7 The expression of miR-214-3p in osteocytes from OC-miR-214 mice and the WT controls. Real-Time PCR of the levels of osteocytic miR-214-3p in OC-miR-214-3p mice and WT controls, respectively. The osteocyte RNA were extracted from the tibia diaphysis after sequential digestions in 0.2% type 1 collagenase / 0.05% trypsin solution, 0.53mM EDTA / 0.05% trypsin solution and 0.2% collagenase / 0.05% trypsin solution at 37°C. *n*=6 for each group. Data are the mean ± s.d.

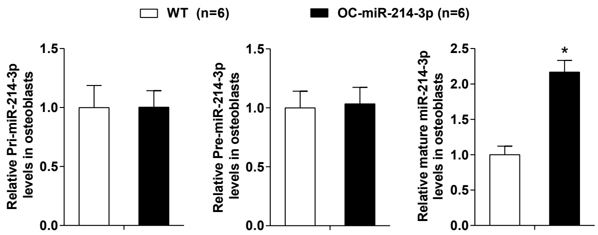

Supplementary Figure 8 The expression of pri-miR-214-3p, pre-miR-214-3p and mature miR-214-3p in osteoblasts in OC-miR-214-3p mice. Real-time PCR analysis of the levels of pri-miR-214-3p, pre-miR-214-3p and mature miR-214-3p in osteoblasts (ALP$^+$ cells) isolated from bone marrow cells by fluorescence-activated cell sorting (FACS) in WT and OC-miR-214-3p mice. The *n* value for each group is indicated at the top of the histograms. All data are the mean ±s.d. *P < 0.05. Student's *t* test was performed.

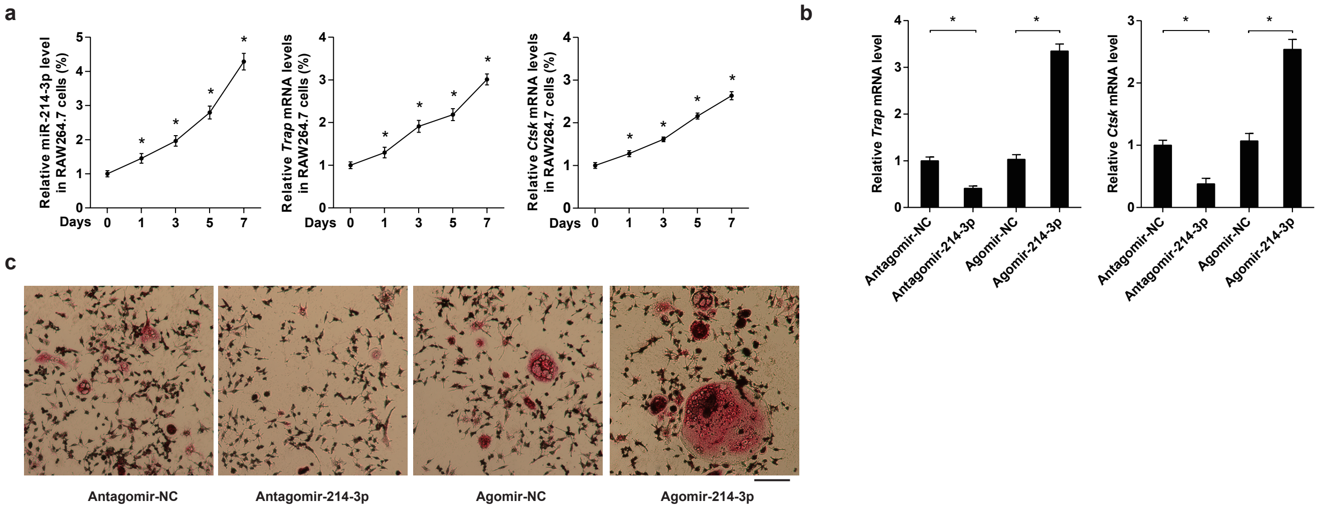

Supplementary Figure 9 miR-214-3p promotes osteoclast differentiation *in vitro*. (**a**) Real-time PCR analysis of the levels of miR-214-3p (left) and mRNA of *Trap* and *Ctsk* (right) in RAW264.7 cells at day 1, 3, 5 and 7 after induction by M-CSF (30 ng/ml) and RANKL (5 ng/ml), respectively. *P < 0.05 *vs.* previous time point. One-way ANOVA with a *post-hoc* test was performed. (**b**) Real-time PCR analysis of *Trap* and *Ctsk* mRNA levels in RAW264.7 cells after treatment of antagomir-214-3p or agomir-214-3p or their corresponding negative controls, respectively. *P < 0.05. One-way ANOVA with a *post-hoc* test was performed. (**c**) Representative micrographs of TRAP$^+$ osteoclasts after treatment with antagomir-214-3p or agomir-214-3p. Scale bar, 50 μm. Data are the mean ± s.d. of 3 independent experiments.

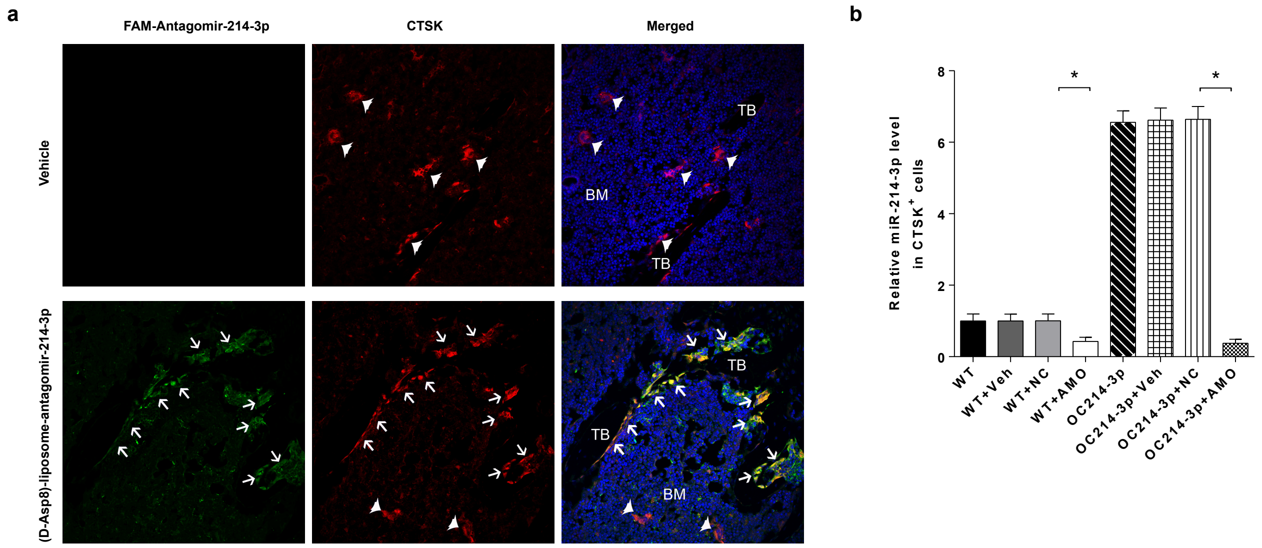

Supplementary figure 10 Cellular distribution of (D-Asp8)-liposome-antagomir-214-3p and miR-214-3p levels after rescue treatment *in vivo*. (**a**) Representative confocal images of the distal femur cryosections from mice after intravenous injection of vehicle (upper panel) or (D-Asp_8)-liposome-antagomir-214 (lower panel). The antagomir-214 was labelled with FAM (green, left column). Immunofluorescence staining was performed to detect the CTSK $^+$ osteoclasts (red, middle column). Merged images with DAPI staining showing the co-localization of antagomir-214 with CTSK $^+$ osteoclasts (right column). Scale bars, 100 μm. Arrows indicate CTSK $^+$ cells with co-localization of FAM-antagomir-214. Arrow heads indicate CTSK $^+$ cells without co-localization of FAM-antagomir-214. (**b**) Real-time PCR analysis of the miR-214-3p levels in CTSK$^+$ cells isolated from distal femur cryosections by laser-captured microdissection in OC-miR-214-3p or WT mice after treatment with PBS, Veh, NC or AMO. *n*=5~7 for each group. Data are the mean ± s.d. *P<0.05. One-way ANOVA with a *post-hoc* test was performed.

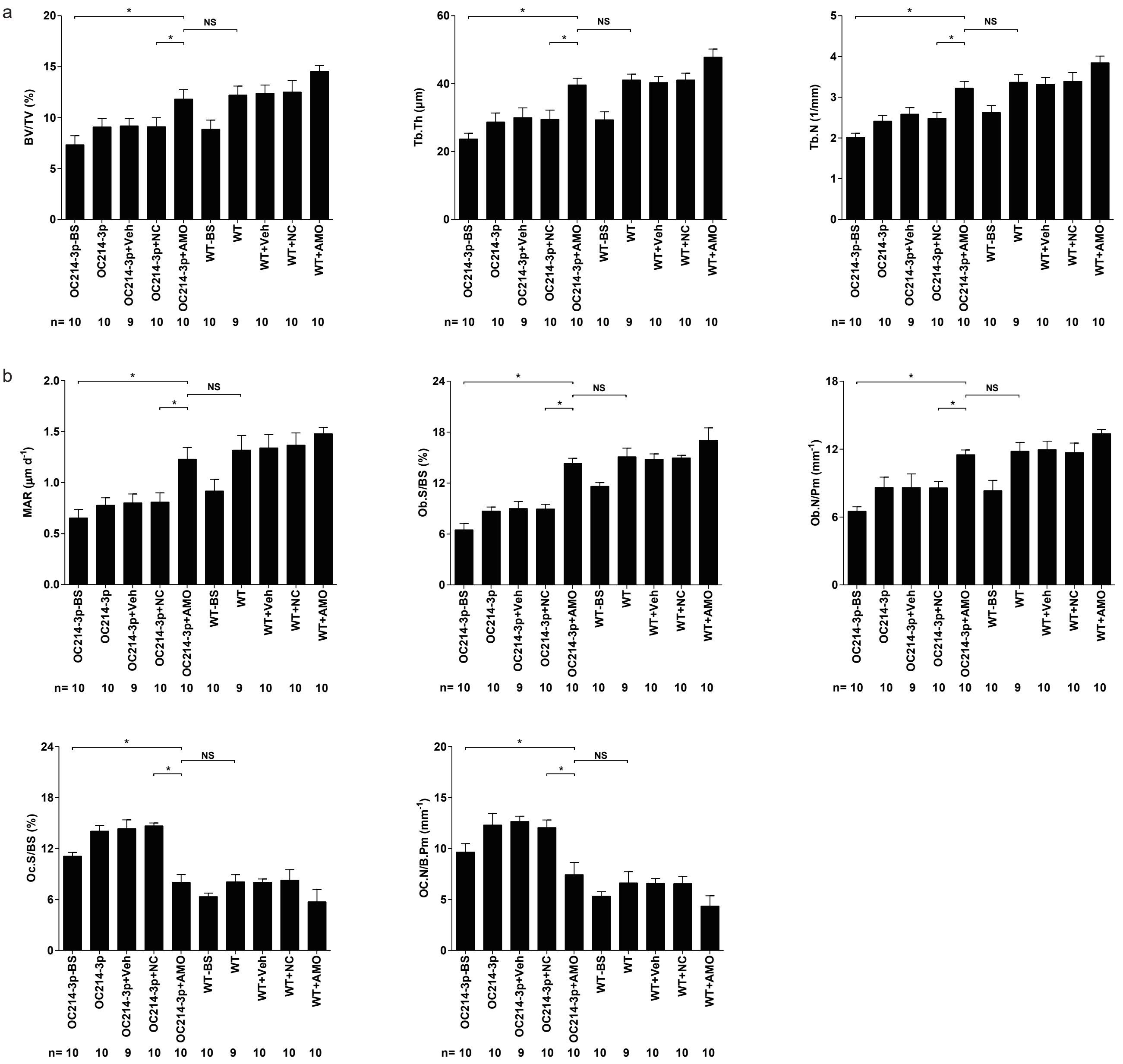

Supplementary Figure 11 The effect of osteoclast-targeted antagomiR-214-3p treatment on bone formation in OC-miR-214-3p mice. (**a**) The value of micro-CT parameters (BV/TV, Tb.Th and Th.N) at the distal femur metaphysis in each group. (**b**) The value of bone histomorphometry parameters (MAR, Ob.S/BS, Ob.N/B.Pm, Oc.S/BS and Oc.N/B.Pm) at the distal femur metaphysis in each group. OC214-3p/WT: OC-miR-214/WT mice treated with PBS, OC214-3p/WT-BS: age-matched OC-miR-214-3p or WT mice that were sacrificed before treatment as baseline, OC214-3p/WT+Veh: OC-miR-214-3p or WT mice treated with (D-Asp)$_8$-liposome (vehicle) alone, OC214-3p/WT+NC: OC-miR-214-3p or WT mice treated with (D-Asp)$_8$- liposome-antagomir nonsense control, OC214-3p/WT+AMO: OC-miR-214-3p or WT mice treated with (D-Asp)$_8$- liposome-antagomir-214-3p. *n*=9~10 for each group. Data are the means ± s.d. **P*<0.05. NS, not significant. One-way ANOVA with a *post-hoc* test was performed.

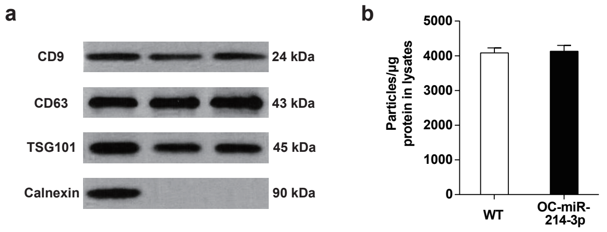

Supplementary Figure 12 Characterization of exosomes isolated from the supernatant in the co-culture systems. (**a**) Western blot analysis of the exosome proteins (CD9, CD63 and TSG101) and Calnaxin in supernatant extracts from OC-miR-214-3p or WT osteoclasts. (**b**) The number of particles assessed by Nanosight technology after normalization with the total amount of protein of cell lysates from the above supernatant. Data are the mean ± s.d. of 4 independent experiments.

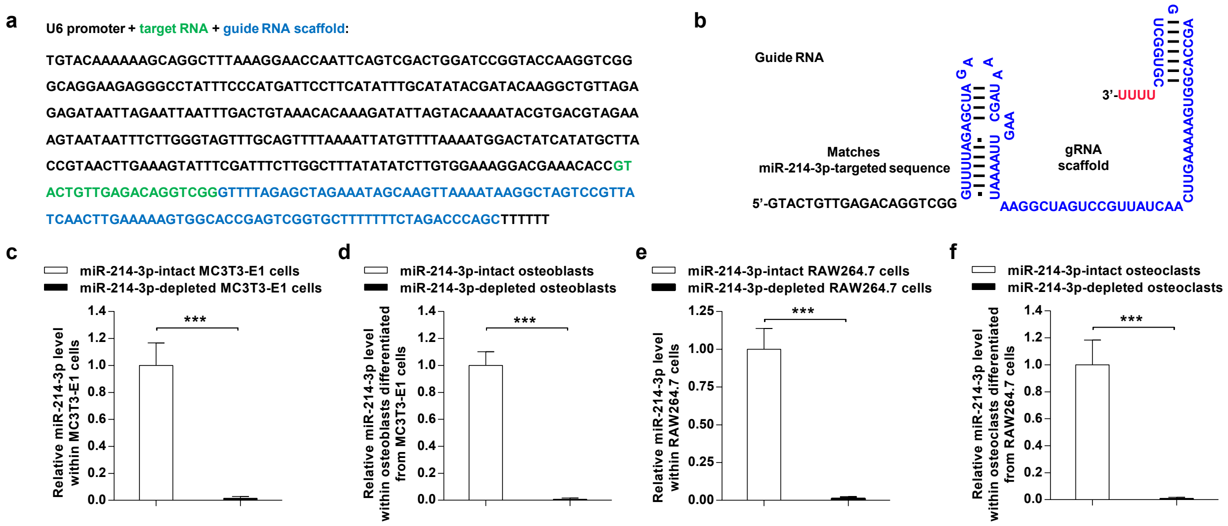

Supplementary Figure 13 RNA-guided genome editing of miR-214-3p locus using the engineered CRISPR-Cas9 system in MC3T3-E1 cells and RAW264.7 cells. (**a**) A U6 promoter based expression scheme for the guide RNAs targeted miR-214-3p locus and (**b**) predicted RNA transcript secondary structure. (**c**) Real-time PCR analysis of the miR-214-3p levels in either miR-214-3p-depleted MC3T3-E1 cells after miR-214-3p gene editing by CRISPR-Cas system or miR-214-3p-intact MC3T3-E1 cells. (**d**) Real-time PCR analysis of the miR-214-3p levels in osteoblasts differentiated from either miR-214-3p-depleted MC3T3-E1 cells or miR-214-3p-intact MC3T3-E1 cells in osteogenic medium. (**e**) Real-time PCR analysis of the miR-214-3p levels in either miR-214-3p-depleted RAW264.7 cells after miR-214-3p gene editing by CRISPR-Cas system or miR-214-3p-intact RAW264.7 cells. (**f**) Real-time PCR analysis of the miR-214-3p levels in osteoclasts differentiated from either miR-214-3p-depleted RAW264.7 cells or miR-214-3p-intact RAW264.7 cells in osteoclastogenic medium. All data are the mean ± s.d. of 3 independent experiments. ***P<0.001 *vs.* WT. Student's t test was performed.

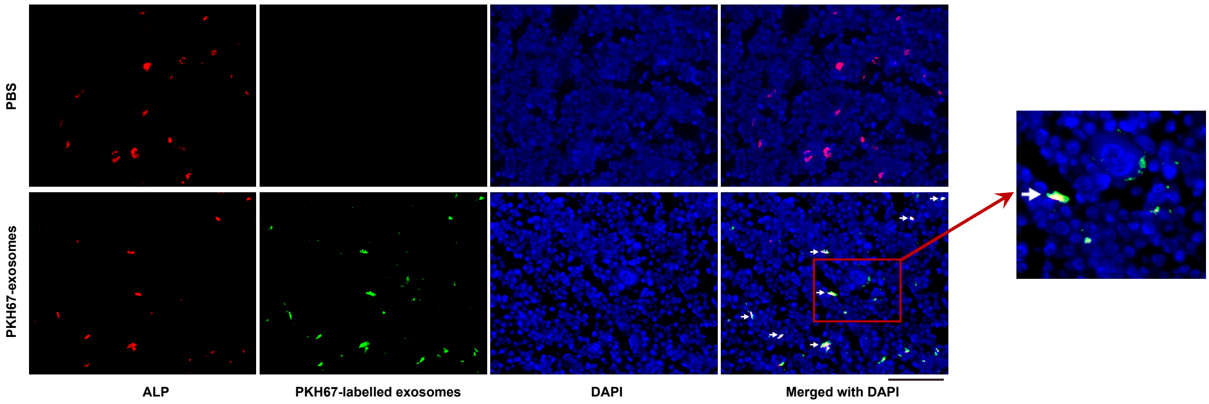

Supplementary Figure 14 Uptake of PKH67-labelled exosomes by osteoblasts *in vivo*. Representative fluorescence micrographs of the distal femur cryosections from the mice at 8 h after intravenously injection with either PBS (upper panel) or PKH67-exosomes (lower panel). Immunofluorescence staining was performed to detect ALP^+ osteoblasts (red, left column). The exosomes were labelled with PKH67 (green, middle left column). The cell nuclei were stained by DAPI (Blue, middle right column). Merged images with DAPI staining showing the co-localization (white arrows) of antagomir-214 with upper panel (yellow color, right column). Scale bars, 100 μm.

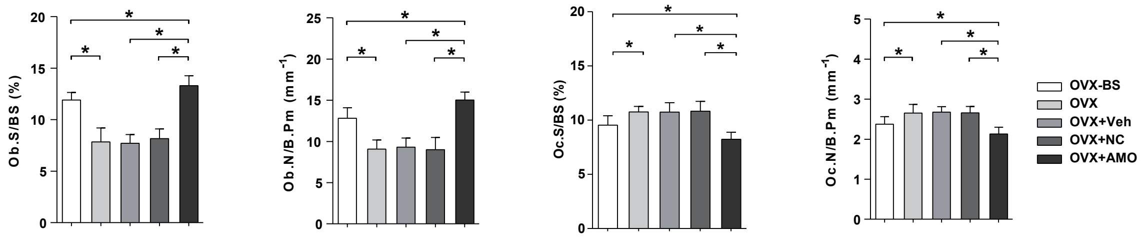

Supplementary Figure 15 The effect of osteoclast-targeted antagomir-214-3p treatment on bone formation in aging OVX mice. The values of bone histomorphometry parameters (Ob.S/BS and Ob.N/B.Pm, Oc.S/BS and Oc.N/B.Pm) at the distal femur metaphysis from mice in each group. OVX-BS: mice sacrificed at 6 months after OVX as baseline, OVX: mice administrated with PBS (negative control), OVX+Veh: mice administrated with delivery system only, OVX+NC: mice administrated with antagomir nonsense control with delivery system, OVX+AMO: mice administrated with (D-Asp$_8$)-liposome-antagomir-214-3p. *n*=9~10 for each group. Data are the mean ± s.d. *P<0.05. One-way ANOVA with a *post-hoc* test was performed.

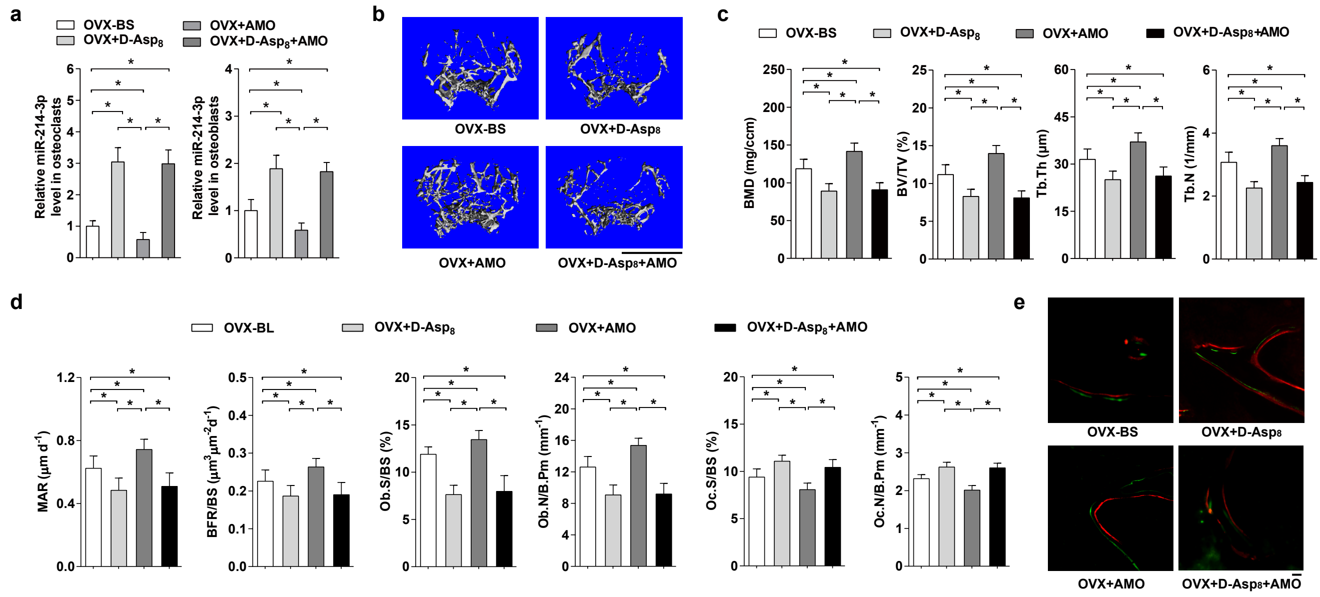

Supplementary figure 16 Pretreatment with D-Asp$_8$ prevents the beneficial effect of osteoclast-targeted antagomir-214-3p treatment on bone formation in aging ovariectomized mice. (**a**) Real-time PCR analysis of the miR-214-3p levels in osteoclasts (*Ctsk*$^+$ cells) and osteoblasts (*Alp*$^+$ cells) isolated from distal femur cryosections by lacer-captured microdissection from mice in each group. (**b**) Representative micro-CT images of the distal femur metaphysis from the mice in each group. Scale bars: 1 mm. (**d**) The values of micro-CT parameters (BMD, BV/TV, Tb.Th and Tb.N) at the distal femur metaphysis from the mice in each group. **(e)** Representative images of new bone formation assessed by double labeling with calcein green and xylenol orange at the distal femur metaphysis from the mice in each group. Scale bars: 10 µm. **(f)** The values of bone histomorphometry parameters (MAR, BFR/BS, Ob.S/BS, Ob.N/B.Pm, Oc.S/BS and Oc.N/B.Pm) at the distal femur metaphysis from the mice in each group. OVX-BS: mice sacrificed at 6 months after OVX as baseline, OVX+D-Asp$_8$: mice administrated with D-Asp$_8$ alone (negative control), OVX+AMO: mice pretreated with PBS 24 hours ahead of administration with (D-Asp$_8$)-liposome-antagomir-214-3p, OVX+D-Asp$_8$+AMO: mice pretreated with D-Asp$_8$ 24 hours ahead of administration with (D-Asp$_8$)-liposome-antagomir-214-3p. *n*=6~8 mice for each group. All data are the mean ± s.d. *P<0.05. One-way ANOVA with a *post-hoc* test was performed.

Western blot scans

Figure 1d

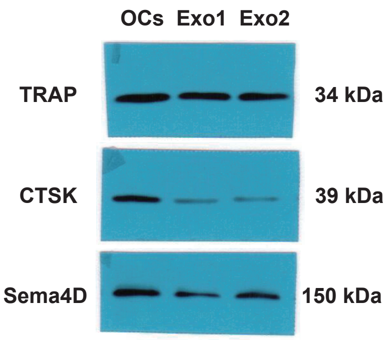

Figure 7c

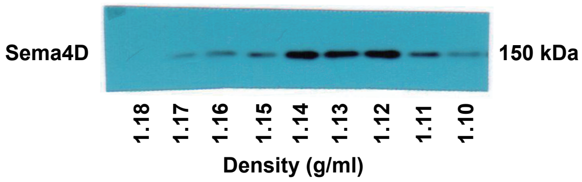

Supplementary figure 1b

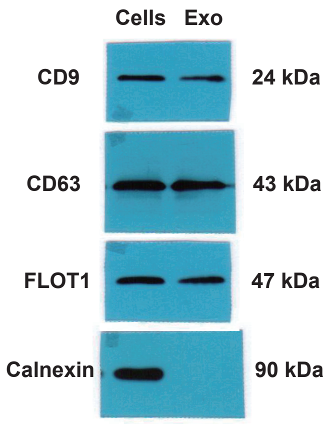

Supplementary figure 9a

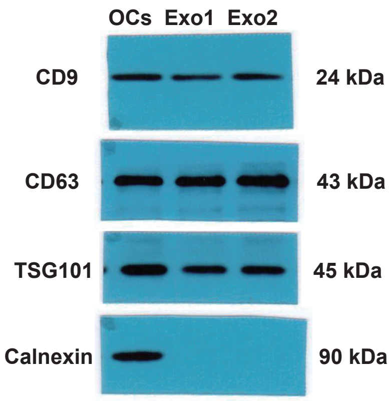

Note: Exo1, exosomes from the supernatant OC-miR-214-3p osteoclasts. Exo2, exosomes from the supernatant of WT osteoclasts.

Supplementary figure 17 The uncropped western blot scans.

Supplementary Table 1 twelve miRNAs examined in whole serum, serum exosomes and bone specimens

miRNA name	Sequence	Accession
hsa-miR-214-3p	ACAGCAGGCACAGACAGGCAGU	MIMAT0000271
mmu-miR-214-3p	ACAGCAGGCACAGACAGGCAGU	MIMAT0000661
hsa-miR-125b-5p	UCCCUGAGACCCUAACUUGUGA	MIMAT0000423
mmu-miR-125b-5p	UCCCUGAGACCCUAACUUGUGA	MIMAT0000136
hsa-miR-133b	UUUGGUCCCCUUCAACCAGCUA	MIMAT0000770
mmu-miR-133b	UUUGGUCCCCUUCAACCAGCUA	MIMAT0000769
hsa-miR-135a-5p	UAUGGCUUUUUAUUCCUAUGUGA	MIMAT0000428
mmu-miR-135a-5p	UAUGGCUUUUUAUUCCUAUGUGA	MIMAT0000147
hsa-miR-29a-3p	UAGCACCAUCUGAAAUCGGUUA	MIMAT0000086
mmu-miR-29a-3p	UAGCACCAUCUGAAAUCGGUUA	MIMAT0000535
hsa-miR-29b-3p	UAGCACCAUUUGAAAUCAGUGUU	MIMAT0000100
mmu-miR-29b-3p	UAGCACCAUUUGAAAUCAGUGUU	MIMAT0000127
hsa-miR-29c-3p	UAGCACCAUUUGAAAUCGGUUA	MIMAT0000681
mmu-miR-29c-3p	UAGCACCAUUUGAAAUCGGUUA	MIMAT0000536
hsa-miR-141-3p	UAACACUGUCUGGUAAAGAUGG	MIMAT0000432
mmu-miR-141-3p	UAACACUGUCUGGUAAAGAUGG	MIMAT0000153
hsa-miR-199a-5p	CCCAGUGUUCAGACUACCUGUUC	MIMAT0000231
mmu-miR-199a-5p	CCCAGUGUUCAGACUACCUGUUC	MIMAT0000229
hsa-miR-200a-3p	UAACACUGUCUGGUAACGAUGU	MIMAT0000682
mmu-miR-200a-3p	UAACACUGUCUGGUAACGAUGU	MIMAT0000519
hsa-miR-206	UGGAAUGUAAGGAAGUGUGUGG	MIMAT0000462
mmu-miR-206	UGGAAUGUAAGGAAGUGUGUGG	MIMAT0000239
hsa-miR-208a-3p	AUAAGACGAGCAAAAAGCUUGU	MIMAT0000241
mmu-miR-208a-3p	AUAAGACGAGCAAAAAGCUUGU	MIMAT0000520

Supplementary Table 2 Clinical features of elderly patients with fractures involved in bone specimens' analysis

Patient	Age	Gender	T score for BMD at Lumbar Spine	Serum BGLAP	Diagosis	Surgical operation
1	61	Female	-1.11	22.8	Proximal Femur Fracture (Left)	Total Hip Replacement
2	62	Female	-1.23	21.9	Femoral Neck Fracture (Left)	Total Hip Replacement
3	63	Female	-1.18	22.3	Proximal Femur Fracture (Right)	Femoral Head Replacement
4	64	Female	-1.33	21.6	Proximal Femur Fracture (Left)	Total Hip Replacement
5	64	Female	-1.39	21.1	Femoral Neck Fracture (Right)	Total Hip Replacement
6	65	Female	-1.35	21.4	Proximal Femur Fracture (Right)	Femoral Head Replacement
7	66	Female	-1.42	19.8	Femoral Neck Fracture (Right)	Total Hip Replacement
8	67	Female	-1.46	20.4	Proximal Femur Fracture (Right)	Total Hip Replacement
9	67	Female	-1.45	20.7	Femoral Neck Fracture (Left)	Femoral Head Replacement
10	68	Female	-1.48	19.7	Proximal Femur Fracture (Right)	Total Hip Replacement
11	69	Female	-1.52	19.2	Femoral Neck Fracture (Left)	Total Hip Replacement
12	70	Female	-1.49	19.6	Femoral Neck Fracture (Right)	Total Hip Replacement
13	71	Female	-1.53	19.3	Proximal Femur Fracture (Right)	Total Hip Replacement
14	72	Female	-1.51	19.4	Femoral Neck Fracture (Right)	Femoral Head Replacement
15	72	Female	-1.62	18.1	Femoral Neck Fracture (Left)	Total Hip Replacement
16	73	Female	-1.47	19.0	Femoral Neck Fracture (Right)	Total Hip Replacement
17	74	Female	-1.66	17.8	Proximal Femur Fracture (Left)	Total Hip Replacement
18	75	Female	-1.63	18.3	Femoral Neck Fracture (Right)	Femoral Head Replacement
19	76	Female	-1.59	19.2	Proximal Femur Fracture (Right)	Total Hip Replacement
20	76	Female	-1.65	17.5	Proximal Femur Fracture (Left)	Femoral Head Replacement

21	77	Female	-1.71	17.4	Proximal Femur Fracture (Right)	Femoral Head Replacement
22	78	Female	-1.72	16.8	Femoral Neck Fracture (Right)	Total Hip Replacement
23	78	Female	-1.68	17.6	Femoral Neck Fracture (Left)	Total Hip Replacement
24	78	Female	-1.74	16.9	Proximal Femur Fracture (Left)	Total Hip Replacement
25	79	Female	-1.75	16.2	Proximal Femur Fracture (Right)	Femoral Head Replacement
26	79	Female	-1.72	16.5	Femoral Neck Fracture (Right)	Total Hip Replacement
27	80	Female	-1.76	15.8	Proximal Femur Fracture (Right)	Femoral Head Replacement
28	81	Female	-1.77	15.6	Femoral Neck Fracture (Right)	Total Hip Replacement
29	82	Female	-1.79	14.9	Proximal Femur Fracture (Right)	Total Hip Replacement
30	83	Female	-1.80	16.2	Proximal Femur Fracture (Right)	Total Hip Replacement
31	83	Female	-1.78	15.4	Femoral Neck Fracture (Left)	Femoral Head Replacement
32	84	Female	-1.82	13.6	Femoral Neck Fracture (Right)	Femoral Head Replacement
33	84	Female	-1.83	14.3	Proximal Femur Fracture (Right)	Total Hip Replacement
34	85	Female	-1.79	13.8	Femoral Neck Fracture (Right)	Total Hip Replacement
35	85	Female	-1.81	12.9	Femoral Neck Fracture (Left)	Total Hip Replacement
36	86	Female	-1.80	13.4	Femoral Neck Fracture (Left)	Femoral Head Replacement
37	87	Female	-1.84	13.2	Proximal Femur Fracture (Right)	Total Hip Replacement
38	88	Female	-1.89	12.4	Proximal Femur Fracture (Right)	Total Hip Replacement
39	88	Female	-1.85	13.1	Femoral Neck Fracture (Right)	Total Hip Replacement
40	89	Female	-1.88	11.7	Proximal Femur Fracture (Right)	Femoral Head Replacement

Supplementary Table 3 Clinical features of age-matched patients without fractures involved in bone specimens' analysis

Patient	Age	Gender	T score for BMD at Lumbar Spine	Serum BGLAP	Diagosis	Site	Surgical operation
1	62	Female	-0.79	25.1	Repetitive dislocation	Right hip	Revision total hip arthroplasty
2	62	Female	-0.84	24.6	Implant loosening	Right hip	Revision total hip arthroplasty
3	63	Female	-0.91	22.9	Implant loosening	Left hip	Revision total hip arthroplasty
4	64	Female	-0.97	22.5	Infection	Left knee	Revision total knee arthroplasty
5	65	Female	-1.03	23.8	Repetitive dislocation	Right hip	Revision total hip arthroplasty
6	65	Female	-1.01	21.6	Infection	Left knee	Revision total knee arthroplasty
7	67	Female	-1.14	20.8	Implant loosening	Right knee	Revision total knee arthroplasty
8	69	Female	-1.19	22.5	Repetitive dislocation	Right hip	Revision total hip arthroplasty
9	70	Female	-1.38	19.9	Repetitive dislocation	Left hip	Revision total hip arthroplasty
10	71	Female	-1.33	19.6	Implant loosening	Right knee	Revision total knee arthroplasty
11	72	Female	-1.41	20.3	Implant loosening	Left knee	Revision total knee arthroplasty
12	74	Female	-1.42	19.3	Implant loosening	Left hip	Revision total hip arthroplasty
13	75	Female	-1.37	18.2	Infection	Right knee	Revision total knee arthroplasty
14	77	Female	-1.52	18.5	Repetitive dislocation	Right hip	Revision total hip arthroplasty
15	78	Female	-1.53	18.9	Implant loosening	Left hip	Revision total hip arthroplasty
16	81	Female	-1.57	17.9	Implant loosening	Left knee	Revision total knee arthroplasty
17	82	Female	-1.61	16.7	Implant loosening	Right hip	Revision total hip arthroplasty
18	83	Female	-1.58	16.8	Implant loosening	Right hip	Revision total hip arthroplasty
19	84	Female	-1.65	15.8	Infection	Right knee	Revision total knee arthroplasty
20	87	Female	-1.72	15.6	Implant loosening	Right knee	Revision total knee arthroplasty
21	89	Female	-1.74	15.5	Infection	Left hip	Revision total hip arthroplasty

Supplementary Table 4 Raw CT for Figure 1b

CT value	Serum				Serum exosomes				Bone specimens			
	Age Mat		Elderly Pat		Age Mat		Elderly Pat		Age Mat		Elderly Pat	
	214	U6	214	U6	214	U6	214	U6	214	U6	214	U6
60-69	23.5	22.6	22.7	22.2	25.9	24.9	24.7	24.1	19.1	18.2	20.7	18.2
	24.4	22.2	22.6	22.1	26.6	24.4	25.3	24.2	19.9	18.2	19.5	17.9
	22.7	22.5	22.3	22.5	24.6	24.4	24.1	24.2	18.7	18.1	19.0	18.0
	22.5	22.9	22.4	22.1	24.0	24.5	25.2	24.2	18.0	17.9	19.3	18.0
	23.3	22.4	22.0	22.1	25.0	24.2	24.3	24.4	19.2	17.9	19.2	18.3
	23.2	22.4	22.6	22.3	25.3	24.5	24.7	24.5	19.7	17.6	19.5	17.9
	22.2	22.4	22.0	22.4	24.7	24.9	23.6	24.2	18.0	18.0	18.5	18.2
	22.5	22.8	21.6	22.4	23.8	24.1	23.8	24.7	17.5	17.4	18.0	18.1
			22.4	22.7			24.6	24.3			18.2	18.5
			21.6	22.5			24.2	24.9			17.6	18.2
			21.3	22.3			23.2	24.6			17.6	18.1
70-69	21.8	22.2	21.0	22.1	24.3	24.8	23.4	24.6	17.9	18.0	17.1	18.0
	22.8	22.5	21.1	22.5	24.5	24.2	23.3	24.2	18.2	17.6	16.9	18.1
	22.0	22.1	21.3	22.9	24.3	24.5	23.3	24.4	18.0	17.6	17.1	18.1
	21.3	22.3	21.4	23.0	23.2	24.2	22.8	24.4	18.1	17.9	16.3	17.8
	21.4	22.2	20.8	22.6	23.6	24.4	22.5	24.1	17.2	18.0	16.6	18.2
	22.8	23.0	20.9	22.2	23.9	24.2	23.6	24.5	17.9	18.2	16.0	17.6
	21.9	22.6	20.6	22.4	23.5	24.3	23.1	24.9	17.8	18.5	16.3	18.2
			20.4	22.4			23.5	25.0			16.1	17.9
			21.1	22.7			22.7	24.4			15.6	17.9
			20.1	22.4			22.7	24.7			16.0	18.1
			20.2	22.4			22.9	24.8			16.3	18.1
			19.9	22.2			22.4	24.8			16.0	18.2
			20.3	22.4			22.7	24.8			16.3	18.5
			20.6	22.7			22.6	24.4			15.8	18.1
			20.1	22.8			22.2	24.4			15.8	18.1
80-89	21.5	22.7	19.8	22.4	23.2	24.5	21.8	24.2	17.4	18.3	15.7	17.8
	21.1	22.2	20.1	22.4	23.6	24.8	22.1	24.5	17.5	18.0	15.5	17.8
	21.7	22.5	20.5	22.7	23.4	24.2	22.9	24.7	17.0	18.1	15.8	18.1
	21.8	22.6	20.0	22.7	23.4	24.2	22.0	24.9	17.8	18.2	15.5	18.0
	21.7	22.4	19.8	22.5	23.9	24.7	21.6	24.4	17.4	18.5	15.7	18.2
	20.9	22.5	20.4	22.5	23.0	24.6	23.1	24.8	17.4	18.0	15.9	18.5
			19.8	22.6			21.5	24.2			16.0	18.6
			19.6	22.6			22.0	24.5			15.3	18.0
			19.6	22.6			21.5	24.6			15.1	17.8
			20.1	22.7			22.0	24.6			15.2	17.9

	19.5	22.4	21.6	24.6	15.1	17.9
	20.2	22.8	22.6	24.7	15.4	18.2
	19.1	22.2	21.9	24.7	15.7	18.6
	19.5	22.5	21.6	24.4	15.7	18.8

Note: Age Mat, age-matched patients without fracture. Elderly Pat, elderly patients with fracture. 214, miR-214-3p. U6, RUN6B.

Supplementary Table 5 Raw data for real time-PCR

Raw data for Figure 1b

Groups			60-69	70-79	80-89
Serum miR-214-3p	Age-matched patients without fracture	$2^{-\Delta CT}$	0.80±0.40	1.37±0.38	2.05±0.49
	Elderly patients with fracture	$2^{-\Delta CT}$	1.25±0.47	3.76±01.19	6.52±1.35
Exosomal miR-214-3p	Age-matched patients without fracture	$2^{-\Delta CT}$	0.83±0.42	1.42±0.40	2.13±0.51
	Elderly patients with fracture	$2^{-\Delta CT}$	1.19±0.66	3.28±1.01	5.97±1.55
Intra-osseous miR-214-3p	Age-matched patients without fracture	$2^{-\Delta CT}$	0.61±0.30	1.16±0.43	1.72±0.35
	Elderly patients with fracture	$2^{-\Delta CT}$	0.77±0.48	3.59±1.06	6.27±1.02

Raw data for Figure 1c

Groups			60-69	70-79	80-89
BGLAP/ GAPDH	Elderly patients with fracture	$2^{-\Delta CT}$	0.94±0.21	0.53±0.21	0.19±0.14

Raw data for Figure 1g

Groups		OVX-6mon-BS	OVX-9mon	OVX-12mon
Serum miR-214-3p	$2^{-\Delta CT}$	0.86±0.32	3.40±1.07	6.31±1.06
Exosomal miR-214-3p	$2^{-\Delta CT}$	0.88±0.33	3.29±1.43	7.04±1.96
Osteoclastic pri-miR-214-3p	$2^{-\Delta CT}$	2.19±0.51	3.42±0.49	4.43±0.59
Osteoclastic pre-miR-214-3p	$2^{-\Delta CT}$	3.21±0.82	6.50±1.06	10.29±1.56
Osteoclastic miR-214-3p	$2^{-\Delta CT}$	4.50±2.79	12.90±3.79	21.65±5.65

Raw data for Figure 2c-1

Groups		Wild type		OC-miR-214-3p	
		OCs	Non-OCs	OCs	Non-OCs
Pri-miR-214-3p	$2^{-\triangle CT}$	1.95±0.51	0.57±0.10	9.30±0.77	0.61±0.15
Pre-miR-214-3p	$2^{-\triangle CT}$	3.15±0.75	0.92±0.11	18.43±1.96	0.93±0.19
miR-214-3p	$2^{-\triangle CT}$	4.79±1.13	1.58±0.21	31.94±2.84	2.66±0.30

Raw data for Figure 2c-2

Groups		Wild type	OC-miR-214-3p
Serom exosomal miR-214-3p	$2^{-\triangle CT}$	0.82±0.20	4.10±0.74

Raw data for Figure 2d

Groups		Wild type	OC-miR-214-3p
ALP mRNA	$2^{-\triangle CT}$	0.120±0.018	0.053±0.011
OPN mRNA	$2^{-\triangle CT}$	0.410±0.065	0.215±0.038
BSP mRNA	$2^{-\triangle CT}$	0.050±0.006	0.031±0.008
BGLAP mRNA	$2^{-\triangle CT}$	0.150±0.022	0.084±0.013

Raw data for Figure 3a

Groups		No OC	WT	OC-miR-214-3p
miR-214-3p in OC supernatant	$2^{-\triangle CT}$	0.24±0.04	2.79±0.32	7.75±0.43
Exosomal miR-214-3p in OC supernatant	$2^{-\triangle CT}$	0.30±0.06	3.25±0.25	9.07±0.39
miR-214-3p in osteoblasts	$2^{-\triangle CT}$	0.40±0.06	0.88±0.05	2.46±0.07

Raw data for Figure 3b

Groups		No OC	WT	OC-miR-214-3p
Pri-miR-214-3p in osteoblasts	$2^{-\triangle CT}$	0.260±0.038	0.263±0.031	0.251±0.043
Pre-miR-214-3p in osteoblasts	$2^{-\triangle CT}$	0.330±0.042	0.328±0.056	0.325±0.044

Raw data for Figure 3c

Groups		No OC	WT	OC-miR-214-3p
ALP mRNA	$2^{-\triangle CT}$	0.110±0.015	0.069±0.010	0.041±0.008
OPN mRNA	$2^{-\triangle CT}$	0.370±0.054	0.246±0.033	0.123±0.029
BSP mRNA	$2^{-\triangle CT}$	0.049±0.007	0.033±0.006	0.023±0.005
BGLAP mRNA	$2^{-\triangle CT}$	0.140±0.013	0.091±0.016	0.051±0.013

Raw data for Figure 3e

Groups		No OC	WT	OC-miR-214-3p	OC-miR-214-3p+ Veh	OC-miR-214-3p+ 3’UTR-NC	OC-miR-214-3p+ ATF4 mRNA 3’UTR
ALP mRNA	$2^{-\triangle CT}$	0.110±0.015	0.069±0.010	0.041±0.008	0.042±0.006	0.042±0.009	0.068±0.011
OPN mRNA	$2^{-\triangle CT}$	0.370±0.054	0.246±0.033	0.123±0.029	0.126±0.029	0.132±0.032	0.242±0.031
BSP mRNA	$2^{-\triangle CT}$	0.049±0.007	0.033±0.006	0.023±0.005	0.023±0.004	0.023±0.003	0.033±0.006
BGLAP mRNA	$2^{-\triangle CT}$	0.140±0.013	0.091±0.016	0.051±0.013	0.052±0.013	0.053±0.017	0.092±0.008

Raw data for Figure 3i

Groups		No OC	WT OC	OC-miR-214-3p
miR-214-3p	$2^{-\triangle CT}$	0.0013±0.0005	0.38±0.06	1.27±0.14

Raw data for Figure 3j

Groups		No OC	WT osteoclasts	miR-214-3p-depleted OCs
Pri-miR-214-3p in osteoblasts	$2^{-\triangle CT}$	0.23±0.03	0.24±0.04	0.24±0.04
Pre-miR-214-3p in osteoblasts	$2^{-\triangle CT}$	0.31±0.05	0.32±0.05	0.31±0.04
miR-214-3p in osteoblasts	$2^{-\triangle CT}$	0.38±0.05	0.91±0.05	0.52±0.05

Raw data for Figure 4f

Groups		No Exo	OC Exo	Exo+Sema4D	Exo+isotype
Pri-miR-214-3p in osteoblasts	$2^{-\triangle CT}$	0.28±0.04	0.28±0.03	0.28±0.04	0.28±0.04
Pre-miR-214-3p in osteoblasts	$2^{-\triangle CT}$	0.35±0.04	0.35±0.06	0.34±0.05	0.34±0.07
miR-214-3p in osteoblasts	$2^{-\triangle CT}$	0.43±0.05	1.27±0.09	0.63±0.06	1.28±0.08

Raw data for Figure 4g

Groups		WT-Exo	miR-214-3p-Exo
Pri-miR-214-3p in osteoblasts	$2^{-\triangle CT}$	0.32±0.06	0.34±0.005
Pre-miR-214-3p in osteoblasts	$2^{-\triangle CT}$	0.34±0.05	0.35±0.06
miR-214-3p in osteoblasts	$2^{-\triangle CT}$	0.41±0.09	0.98±0.14

Raw data for Figure 4h

Groups		WT-Exo	miR-214-3p-Exo
ALP mRNA	$2^{-\triangle CT}$	0.060±0.009	0.027±0.006
OPN mRNA	$2^{-\triangle CT}$	0.230±0.038	0.119±0.035
BSP mRNA	$2^{-\triangle CT}$	0.030±0.006	0.019±0.005
BGLAP mRNA	$2^{-\triangle CT}$	0.091±0.014	0.050±0.010

Raw data for Figure 5b

Groups		OVX-BL	OVX	OVX+Veh	OVX+NC	OVX+AMO	Sham
miR-214-3p in osteoclasts	$2^{-\triangle CT}$	1.71±0.34	5.23±0.55	5.33±0.59	5.37±0.62	1.05±0.34	0.73±0.29
miR-214-3p in osteoblasts	$2^{-\triangle CT}$	0.16±0.03	0.30±0.03	0.31±0.03	0.30±0.03	0.09±0.02	0.05±0.02

Supplementary Table 6 Primers used for real-time PCR

Gene	Primers (5'-3')	
	Forward	Reverse
Human *BGLAP*	CACTCCTCGCCCTATTGGC	CCCTCCTGCTTGGACACAAAG
Human *GAPDH*	CTGGGCTACACTGAGCACC	AAGTGGTCGTTGAGGGCAATG
Mouse *Atf4*	CCTGAACAGCGAAGTGTTGG	TGGAGAACCCATGAGGTTTCAA
Mouse *Bglap*	TGCTTGTGACGAGCTATCAG	GAGGACAGGGAGGATCAAGT
Mouse *Gapdh*	AGGTCGGTGTGAACGGATTTG	TGTAGACCATGTAGTTGAGGTCA
Mouse *Alp*	CCAACTCTTTTGTGCCAGAGA	GGCTACATTGGTGTTGAGCTTTT
Mouse *Col1a1*	GCTCCTCTTAGGGGCCACT	CCACGTCTCACCATTGGGG
Mouse *Opn*	AGCAAGAAACTCTTCCAAGCAA	GTGAGATTCGTCAGATTCATCCG
Mouse *Bsp*	ATGGAGACGGCGATAGTTCC	CTAGCTGTTACACCCGAGAGT
Mouse *Runx2*	ATGGGACTGTGGTTACCGTCAT	AAGGTGAAACTCTTGCCTCGTC
Mouse *Osx*	AGCGACCACTTGAGCAAACAT	GCGGCTGATTGGCTTCTTCT
Mouse *Trap*	GTGGAAGCCTCTGGAAAATC	CTCCTCCCTCACACCCGTTA
Mouse *Ctsk*	GAAGAAGACTCACCAGAAGCAG	TCCAGGTTATGGGCAGAGATT
Mouse *Rank*	TAATCCAGCAGGGAAGCA	CACAGCCCTCAGAATCCA
Mouse *Oscar*	CCGTGCTGACTTCACACCAA	GGGGTGACAAGGCCACTTTT
Mouse *NFATc1*	AGTCATCGGCGGGAAGAAG	CCATTGGCAGGAAGGTACG
Mouse *Sost1*	CCACAGAGGTCATCCCA	GACACATCTTTGGCGTCATAG
Mouse *Dmp1*	AGTGAGTCATCAGAAGAAAGTCAAGC	CTATACTGGCCTCTGT
